# Electronic Consequences of Ligand Substitution at Heterometal Centers in Polyoxovanadium Clusters: Controlling the Redox Properties through Heterometal Coordination Number

**DOI:** 10.1002/chem.201905624

**Published:** 2020-06-25

**Authors:** Rachel L. Meyer, Montaha H. Anjass, Brittney E. Petel, William W. Brennessel, Carsten Streb, Ellen M. Matson

**Affiliations:** ^1^ Department of Chemistry University of Rochester Rochester NY 14627 USA; ^2^ Institute of Inorganic Chemistry I Ulm University Albert-Einstein-Allee 11 89081 Ulm Germany; ^3^ Helmholtz Institute Ulm (HIU) Helmholtzstrasse 11 89081 Ulm Germany

**Keywords:** coordination chemistry, electrochemistry, metal oxide, polyoxometalate, self-assembly

## Abstract

The rational control of the electrochemical properties of polyoxovanadate‐alkoxide clusters is dependent on understanding the influence of various synthetic modifications on the overall redox processes of these systems. In this work, the electronic consequences of ligand substitution at the heteroion in a heterometal‐functionalized cluster was examined. The redox properties of [V_5_O_6_(OCH_3_)_12_FeCl] (**1‐[V_5_FeCl]**) and [V_5_O_6_(OCH_3_)_12_Fe]X (**2‐[V_5_Fe]X**; X=ClO_4_, OTf) were compared in order to assess the effects of changing the coordination environment around the iron center on the electrochemical properties of the cluster. Coordination of a chloride anion to iron leads to an anodic shift in redox events. Theoretical modelling of the electronic structure of these heterometal‐functionalized clusters reveals that differences in the redox profiles of **1‐[V_5_FeCl]** and **2‐[V_5_Fe]X** arise from changes in the number of ligands surrounding the iron center (e.g., 6‐coordinate vs. 5‐coordinate). Specifically, binding of the chloride to the sixth coordination site appears to change the orbital interaction between the iron and the delocalized electronic structure of the mixed‐valent polyoxovanadate core. Tuning the heterometal coordination environment can therefore be used to modulate the redox properties of the whole cluster.

## Graphical abstract

**Combined experimental and theoretical methods** are used to gain fundamental understanding of the redox‐properties of iron‐vanadium oxo‐alkoxide clusters as they related to modification of the heterometal number.

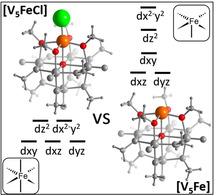

## Introduction

Polyoxometalates (POMs) are molecular metal oxide clusters that have shown great utility in many areas of research, including medicinal chemistry,^1^ material science,^2^ molecular magnetism,^3^ catalysis,^4^ and energy storage.^5^ The rich electrochemical properties and stability exhibited by these polynuclear assemblies make them good candidates for a variety of electrochemical applications. For example, POMs have displayed great promise as electrocatalysts for the reduction of protons and oxoanions (e.g., nitrite, chlorate, and bromate) and the oxidation of alkanes, alkenes, alcohols, sulfoxides, and phosphines.^6^ Recent work has also demonstrated their competency as charge carriers in redox flow batteries.^7^

A popular method for modifying the electrochemical properties of polyoxometalates is to incorporate other elements into the cluster framework. For example, in the case of the Keggin polyoxomolybdate cluster [*X*Mo_12_O_40_]^n−^ (X=P^V^, Si^IV^, etc.), replacement of the central phosphorus atom with silicon shifts the redox events toward more reducing potentials.^8^

While much is known of strategies to modify the redox properties of molybdate and tungstate clusters, considerably less is understood relating to strategies for modulating the electrochemical profiles of vanadium‐oxide derivatives. This is often attributed to the fact that the structural flexibility of vanadium makes precise and controlled modification to polyoxovanadate frameworks challenging.^9^ A few examples of functionalized vanadium oxide clusters in the literature illustrate that, like their molybdate and tungstate congeners, the magnetic, photochemical, electrochemical, and catalytic properties of these assemblies can be tuned.^10^ However, the development of a general theoretical framework linking systematic molecular modifications to the resulting redox behavior displayed by polyoxovanadates has been outside the scope of these studies.

**Figure 1:**
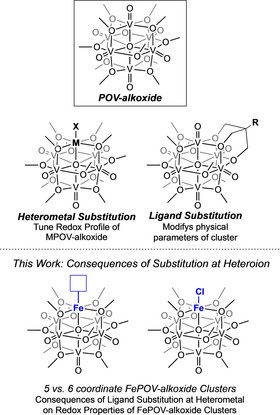
Previously reported synthetic modifications for POV‐alkoxide clusters.^7^, ^13^

Toward building a library of polyoxovanadate clusters with tunable physicochemical properties, part of our research team has been studying the synthesis and characterization of Lindqvist polyoxovanadate‐alkoxide (POV‐alkoxide) clusters, [V_6_O_7_(OR)_12_] (R=CH_3_, C_2_H_5_).^11^ We have demonstrated that the solubility and electrochemical properties of these clusters can be varied through a number of modifications to the cluster core, rendering this family of molecular vanadates useful motifs for probing electrochemical consequences of these structural perturbations (Figure 1). In particular, integration of transition metal or metalloid ions within the cluster core ([V_5_O_6_(OCH_3_)_12_M], M=Ti^4+^, Zr^4+^, Hf^4+^, Fe^3+^, Ga^3+^) has been shown to serve as an effective method for tuning the redox properties of POV‐alkoxides,^12^ similar to previous studies on heterometallic tungstates and molybdates.

While we have provided extensive evidence that the aforementioned variations of heteroion and bridging alkoxide ligand identity can modify the physicochemical properties of the assembly, the effects of modifying the ligand environment surrounding a heteroion embedded within a POV cluster have not been reported. This gap of knowledge is striking when considering the importance of ligand field considerations in dictating the electronic structure of monometallic, transition metal complexes. Herein, we report the electrochemical consequences of halide coordination to the heterometal of an iron‐functionalized POV‐alkoxide cluster, [V_5_O_6_(OCH_3_)_12_FeCl], and comparing its redox properties to that of previously reported, iron‐installed POV‐alkoxide clusters (Figure 1). A combination of experimental and theoretical investigations is used to develop a more complete understanding of the comparative electrochemical effects of incorporating coordinating (e.g., chloride) versus non‐coordinating (e.g., perchlorate) ligands at the site‐differentiated metal center.

## Results and Discussion

### Electrochemical characterization of 1‐[V_5_FeCl]

The isolation of the chloride‐functionalized analogue of the iron‐containing species, [V_5_O_6_(OCH_3_)_12_FeCl] (**1‐[V_5_FeCl]**), was previously reported by some of us, using an adapted synthetic procedure to that reported for the gallium‐functionalized variant, [V_5_O_6_(OCH_3_)_12_GaCl].12d Briefly, self‐assembly of the heterometallic Lindqvist cluster occurs following heating of a tetrahydrofuran solution of [VO(OCH_3_)_3_] (5 equiv.) and FeCl_3_ (2 equiv.) in the presence of NaBH_4_ (1 equiv.). Spectroscopic investigations and charge balancing revealed the oxidation state distribution for complex **1‐[V_5_FeCl]** to be V^IV^_3_V^V^_2_Fe^III^. While the electronic structure of the neutral species was rigorously characterized, the electrochemical profile of **1‐[V_5_FeCl]** was not explored. Thus, we set out to understand the consequences of halide‐coordination to the heteroion in **1‐[V_5_FeCl]** on its redox chemistry and electronic structure.

**Figure 2:**
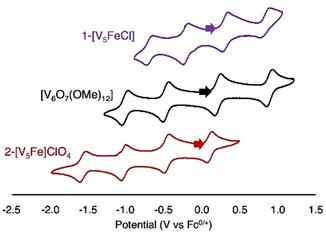
Cyclic voltammograms of **1‐[V_5_FeCl]** (Purple), [V_6_O_7_(OCH_3_)_12_] (Black), and **2‐[V_5_Fe]ClO_4_** (Red) collected in acetonitrile (0.1 m [*n*Bu_4_N][PF_6_] as supporting electrolyte, referenced to Fc^0/+^).

The cyclic voltammogram (CV) of **1‐[V_5_FeCl]**, collected in acetonitrile, displayed four, evenly spaced redox events (E_1/2_=−0.68, −0.21, +0.31, +0.83 V vs. Fc^0/+^; Figure 2); CV experiment with expanded electrochemical window can be found in Figure S1 in the Supporting Information. These events are anodically shifted compared to the hexavanadate cluster by approximately 0.1 V. Thus, the replacement of a “V=O” unit for a “Fe−Cl” effectively modulates the potentials of the redox processes without compromising the rich electrochemical properties. This modulation has also been observed in other heterometal‐functionalized POV‐alkoxides.12c, 12d Interestingly, comparing the redox profile of **1‐[V_5_FeCl]** to other iron‐functionalized POV‐alkoxide clusters reported by our group, namely [V_5_O_6_(OCH_3_)_12_Fe]X (X=ClO_4_, **2‐[V_5_Fe]ClO_4_**; X=OTf, **2‐[V_5_Fe]OTf**), shows that the series of four electrochemical events of the halogenated species is shifted by approximately 1 V. This substantial anodic shift upon coordination of the chloride is unexpected, as the additional negative charge supplied by the anionic ligand should shift the redox profile to more reducing potentials. As such, a more in‐depth chemical analysis of the electronic characteristics of **1‐[V_5_FeCl]** is required to explain this result.

### Isolation of redox isomers of 1‐[V_5_FeCl]

The striking differences in the electrochemical profiles of the two FePOV‐alkoxide clusters, **1‐[V_5_FeCl]** and **2‐[V_5_Fe]X** (X=ClO_4_, OTf), prompted a detailed investigation to understand the changes in the electronic structure of **1‐[V_5_FeCl]** across all charge states. We hypothesized that the change in coordination number of the iron center might be influencing the energetics of electron storage and release in these heterometallic assemblies. Indeed, the neutral complex **1‐[V_5_FeCl]** has been shown to retain a 6‐coordinate iron center (pseudo *O*_h_ geometry) in the solid‐state and in solution.12d In contrast, we have previously reported that the coordination environment of iron in complex **2‐[V_5_Fe]X (X=OTf, ClO_4_)** is best described as square pyramidal (5‐coordinate), as the weakly‐coordinating nature of OTf^−^ and ClO_4_^−^ counter ions results in their dissociation from the cluster core.12a, 12b These conclusions are supported by Mössbauer analysis of solid‐state samples, revealing a mixture of the 5‐coordinate (62 %) and 6‐coordinate (38 %) species in complex **2‐[V_5_Fe]X**. Following cluster reduction, the mixture collapses to a single set of resonances consistent with a 5‐coordinate Fe^III^. Electrospray ionization‐mass spectrometry (ESI‐MS) data also suggests that, in solution, the counter ion (X=OTf, ClO_4_) is fully dissociated from the iron‐functionalized POV‐alkoxide core.

To establish whether the cathodic shift in the redox profile of **1‐[V_5_FeCl]** relative to **2‐[V_5_Fe]X** is a general phenomenon for iron‐functionalized POV‐alkoxide clusters containing 6‐cooridnate iron centers, we obtained the CV of the previously reported complex [V_5_O_6_(OCH_3_)_12_Fe(OCN)], **1‐[V_5_FeOCN]** in acetonitrile (Figure S2, Table S1, CV experiment with expanded electrochemical window in Figure S3). The cyanate derivative was selected because the cyanate anion has been shown to strongly bind to the iron center, and thus is a good example of a 6‐coordinate species.12b The CV of the cyanate‐functionalized cluster is identical to that of **1‐[V_5_FeCl],** providing further support for our hypothesis that the observed ligand‐dependent shifts in redox potentials result from changes in coordination *number* of the iron center, rather than ligand identity.

In particular, we became interested in understanding how the coordination of a chloride ion to the heterometal (iron) affects the storage and release of electron density from the cluster. Synthetic isolation of the various oxidation states of **2‐[V_5_Fe]ClO_4_** and [V_6_O_7_(OR)_12_] (R=CH_3_, C_2_H_5_) has been shown to be an effective method at elucidating the electrochemical properties of these heterometallic systems.11b, 11c, 12b As such, we sought to perform a similar analysis with **1‐[V_5_FeCl]**.

In our original report of the synthesis of **1‐[V_5_FeCl]**, we proposed that the FePOV‐alkoxide cluster bears a mixed‐valent oxidation state distribution of metal ions (V^V^_2_V^IV^_3_Fe^III^).12d The open circuit potential of the neutral cluster (−0.05 V vs. Fc^0/+^) is located between sets of two reduction and oxidation waves. This suggests that the parent cluster **1‐[V_5_FeCl]** could be twice oxidized and twice reduced, affording a family of five redox isomers of the FePOV‐alkoxide cluster, each differing by a single electron.

**Scheme 1:**
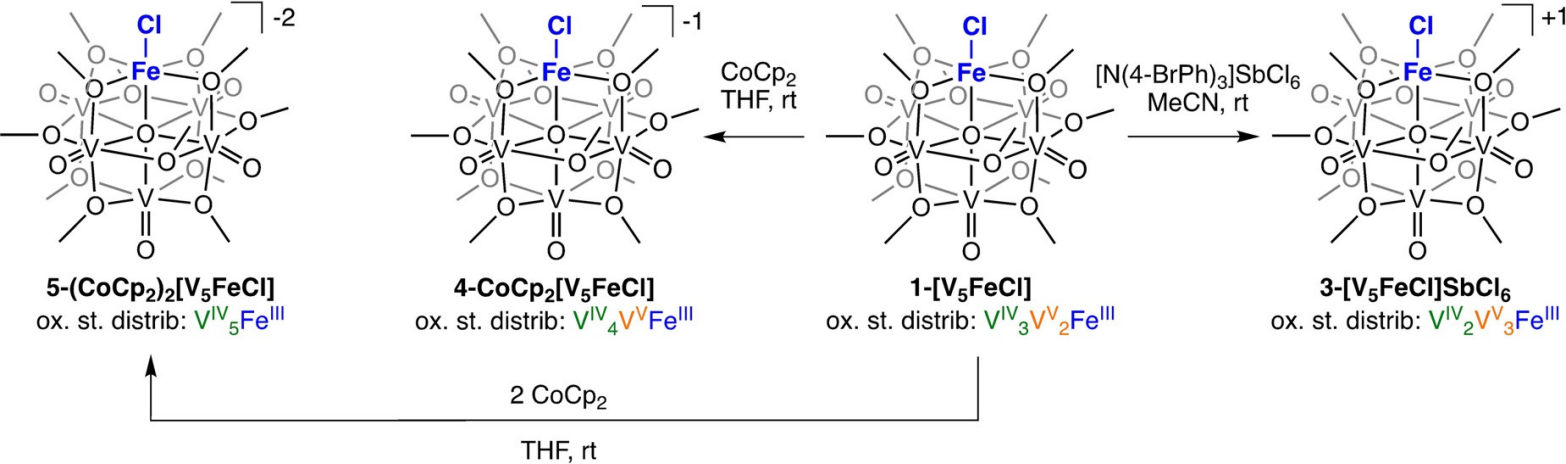
Synthesis and proposed structures of redox isomers of **1‐[V_5_FeCl]**. Synthesis of complexes **3‐[V_5_FeCl]SbCl_6_**, **4‐CoCp_2_[V_5_FeCl]**, and **5‐(CoCp_2_)_2_[V_5_FeCl]**.

Toward isolating the redox series of iron‐functionalized POV‐alkoxide clusters, outlined in Scheme 1, we first set out to synthesize the two products of oxidation. The mono‐cationic cluster, [V_5_O_6_(OCH_3_)_12_FeCl]OTf (**3‐[V_5_FeCl]OTf**), has been previously isolated by addition of a sub‐stoichiometric amount of WCl_6_ to **2‐[V_5_Fe]OTf**.12b To independently synthesize the mono‐cationic species from the iron‐chloride functionalized POV‐alkoxide cluster, an equivalent of (N(C_6_H_4_Br‐4)_3_)SbCl_6_ (E^0^=0.67 V vs. Fc^0/+^ in acetonitrile)^13^ was added to **1‐[V_5_FeCl]** in acetonitrile. The ^1^H NMR spectrum of the product, [V_5_O_6_(OCH_3_)_12_FeCl]SbCl_6_ (**3‐[V_5_FeCl]SbCl_6_**), in CD_3_CN revealed two paramagnetic resonances at 14.5 (24 H) ppm and 12.5 (12 H) ppm (Figure S4). Based on the pseudo‐C_4V_ symmetry of the cluster, one would expect three resonances in the ^1^H NMR spectrum of **3‐[V_5_FeCl]SbCl_6_**. However, a two‐peak pattern with broad resonances that possess a 2:1 ratio suggests that two sets of chemically equivalent methoxide ligands overlap in this spectrum. Similar patterns to those observed in the ^1^H NMR spectrum of **3‐[V_5_FeCl]SbCl_6_** have likewise been reported for other iron‐functionalized, POV‐alkoxide clusters.12a, 12b These signals are substantially shifted from those of the starting material (*δ*=23.3 ppm and 13.3 ppm), suggesting complete conversion to the desired oxidized cluster. Furthermore, the ^1^H NMR spectrum of **3‐[V_5_FeCl]SbCl_6_** collected in [D_8_]THF matches that of previously reported **3‐[V_5_FeCl]OTf** (Figure S5). The product composition was further confirmed by elemental analysis and ESI‐MS (Figure S6).

The CV of complex **1‐[V_5_FeCl]** suggests a second, more‐oxidized product should be accessible *via* the two‐electron oxidation of the parent cluster. However, addition of two equivalents of NOPF_6_ to **1‐[V_5_FeCl]** in dichloromethane (NOPF_6_ in dichloromethane, *E* ^0^=1.00 V vs. Fc^0/+^)^13^ resulted in the formation of the mono‐cationic species, [V_5_O_6_(OCH_3_)_12_FeCl]PF_6_ (confirmed by ^1^H NMR and infrared spectroscopic analyses, Figure S7). Attempts to electrochemically access the di‐cationic species, *via* bulk oxidation in acetonitrile, resulted in decomposition (Figure S8). Indeed, the poor chemical reversibility of this second oxidation event is illustrated when the redox process is isolated at varying scan rates from 10 to 1000 mV s^−1^ in acetonitrile (Figure S9). As the scan rate is increased, the peak‐to‐peak separation between the oxidative and reductive features increases and the redox event becomes ill defined. These results suggest that the most oxidizing event is actually an irreversible process. The inability to synthetically or electrochemically access the di‐cationic species of **1‐[V_5_FeCl]** is not wholly surprising, as we have previously reported the oxidative instability of the homometallic POV‐alkoxide cluster, [V_6_O_7_(OCH_3_)_12_].7d, 11a

In principle, the E_1/2_ value of the most oxidizing event of complex **1‐[V_5_FeCl]** occurs at a potential at which chloride oxidation is possible. To assess whether the irreversibility of this redox event was due to chloride dissociation and subsequent oxidation, we titrated a solution of (*n*Bu_4_N)Cl into a CV cell containing **1‐[V_5_FeCl]** (see supporting information for experimental details, Figure S10). Heterogeneous chloride oxidation results in a distinct electrochemical event at a potential of 0.79 V (vs. Fc^0/+^), as indicated by an increased current response proportional to increased concentrations of (*n*Bu_4_N)Cl in the first scan rate of the sample. Third scan analysis, allowing for the sample to reach chemical equilibrium, reveals chloride consumption, and retention of the four redox processes native to complex **1‐[V_5_FeCl]** (Figure S10), indicating that Cl_2_ production does not correlate with cluster decomposition. As such, we hypothesize that the observed lack of reversibility is indicative of complete cluster degradation, and not dissociation/oxidation of the chloride ligand.

To access the two reduced forms of complex **1‐[V_5_FeCl]** observed by CV, stoichiometric reductions of the neutral FePOV‐alkoxide cluster were performed. Previously, we have reported isolation of a series of reduced, FePOV‐alkoxide clusters resulting from addition of various equivalents of potassium graphite (KC_8_) to **2‐[V_5_Fe]ClO_4_**.12b To mirror these synthetic procedures, one or two equivalents of KC_8_ were added to **1‐[V_5_FeCl]**. In the case of the mono‐reduced derivative, the desired product, K[V_5_O_6_(OCH_3_)_12_FeCl] (**4‐K[V_5_FeCl]**), was isolated in good yield (87 %). Formation of **4‐K[V_5_FeCl]** was verified by ^1^H NMR spectroscopy (23.8 (24 H) and 18.1 (12 H) ppm; Figure S11), ESI‐MS (Figure S12), and elemental analysis.

While the reduction of **1‐[V_5_FeCl]** with one equivalent of KC_8_ cleanly afforded the mono‐reduced cluster, attempts to isolate the di‐reduced species proved challenging. Addition of two equivalents of KC_8_ to **1‐[V_5_FeCl]** resulted in the removal of the chloride ion, as confirmed by ^1^H NMR and infrared spectroscopies (Figure S13). The spectra of the crude reaction compares favorably to that previously reported for the mono‐anionic, iron‐functionalized POV‐alkoxide cluster, K[V_5_O_6_(OCH_3_)_12_Fe].12b We hypothesized that the removal of the chloride ion from the surface of the cluster is promoted by the preferential formation of potassium chloride under highly reducing conditions. Thus, we opted to use a non‐coordinating reductant with an appropriate redox potential to isolate the di‐reduced species. Gratifyingly, the addition of two equivalents of cobaltocene (CoCp_2_, E^0^=−1.33 V vs. Fc^0/+^ in acetonitrile) to **1‐[V_5_FeCl]** afforded the fully‐reduced, heterometallic Lindqvist cluster, (CoCp_2_)_2_[V_5_O_6_(OCH_3_)_12_FeCl] (**5‐(CoCp_2_)_2_[V_5_FeCl]**), in good yield (42 %), as confirmed by ^1^H NMR spectroscopy (Figure S14, *δ*=24.1 and 22.1 ppm) ), ESI‐MS (Figure S15), and elemental analysis. Retention of the chloride‐bound, iron‐functionalized, Lindqvist formulation upon reduction to **5‐(CoCp_2_)_2_[V5FeCl]** was unambiguously confirmed through single crystal X‐ray structural analysis (Figure S16; Table S2). The cobaltocenium derivative of the mono‐reduced cluster, CoCp_2_[V_5_O_6_(OCH_3_)_12_FeCl] (**4‐CoCp_2_[V_5_FeC]**), could also be isolated by reacting one equivalent of CoCp_2_ with **1‐[V_5_FeCl]**. Characterization of **4‐CoCp_2_[V_5_FeCl]** *via* ^1^H NMR (Figure S17) spectroscopy and ESI‐MS (Figure S18) matches that of **4‐K[V_5_FeCl]**. Furthermore, structural analysis of the mono‐reduced species, **4‐CoCp_2_[V_5_FeCl]**, *via* single‐crystal X‐ray diffraction revealed the expected halide‐functionalized Lindqvist structure (Figure S19, Table S2).

### Electronic structure of the redox isomers of 1‐[V_5_FeCl]

With the oxidized and reduced clusters in hand, we turned to elucidating the electronic characteristics of these compounds. In particular, we were interested in understanding the participation of transition metal ions in the reduction and oxidation events observed in the CV of complex **1‐[V_5_FeCl]**. Fourier‐transform infrared (FT‐IR) and electronic absorption spectroscopies are complementary techniques that have been used to give valuable insight into the oxidation state distribution within POV‐alkoxide clusters.11b, 11c, 12b Thus, we applied these spectroscopic methods to analyze the electronic structures of **1‐[V_5_FeCl]**, **3‐[V_5_FeCl]SbCl_6_**, **4‐K[V_5_FeCl]**, and **5‐(CoCp_2_)_2_[V_5_FeCl]**.

**Figure 3:**
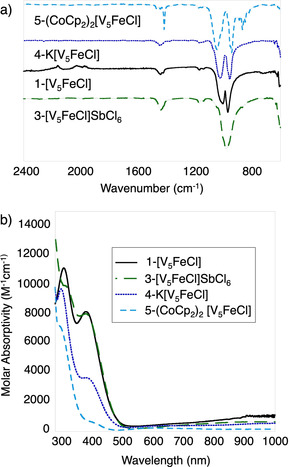
a) infrared spectra and b) electronic absorption spectra (collected in acetonitrile) of **1‐[V_5_FeCl]**, **3‐[V_5_FeCl]SbCl_6_**, **4‐K[V_5_FeCl]**, and **5‐(CoCp_2_)_2_[V_5_FeCl]**.

The FT‐IR spectra of **1‐[V_5_FeCl]**, **4‐K[V_5_FeCl]**, and **5‐(CoCp_2_)_2_[V_5_FeCl]** (Figure 3 a, Table S3) display two intense bands located at 941–969 cm^−1^ and 1043–1003 cm^−1^, corresponding to the terminal oxido, *v*(V*=*O_t_), and the bridging methoxide, *v*(O_b_‐CH_3_), vibrations, respectively. The presence of a singular *v*(V*=*O_t_) and *v*(O_b_‐OCH_3_) band in the FT‐IR spectra of POV‐alkoxide clusters is characteristic of the Robin and Day Class II delocalized electronic structure displayed by these assemblies.11b, 11c Therefore, the FT‐IR spectra of the **1‐[V_5_FeCl]** redox series show that both the Lindqvist motif and the electronic delocalization are retained as electrons are added and removed.

As the cluster is oxidized from the di‐anionic to the neutral species, the *v*(V*=*O_t_) band shifts to higher energy. This is consistent with the strengthening of the terminal V−O bond as electron density is removed from the cluster core. In contrast, the *v*(O_b_‐CH_3_) band shifts to lower energy upon oxidation as a result of the decrease in the partial negative charge on the bridging oxygen atoms. The FT‐IR spectrum of **3‐[V_5_FeCl]SbCl_6_** only displays one broad band at 984 cm^−1^, which is due to the *v*(V*=*O_t_) and *v*(O_b_‐CH_3_) vibrations shifting upon oxidation such that they overlap. Similar shifts in the *v*(V*=*O_t_) and *v*(O_b_‐OCH_3_) bands upon oxidation have been observed in other iron‐functionalized, POV‐alkoxide clusters.12b For these systems, the redox activity was shown to be localized on the vanadium centers, while the Fe^III^ center did not show any redox‐activity across the redox series. The chloride‐functionalized derivatives reported here appear to behave in a similar manner.

Next, electronic absorption spectra of the redox isomers of the halide‐functionalized clusters were collected in acetonitrile (Figure 3 b, Table S4). Complexes **1‐[V_5_FeCl]**, **3‐[V_5_FeCl]SbCl_6_**, and **4‐K[V_5_FeCl]** all have absorbance features at 382 and 990 nm, diagnostic of mixed valent (V^IV^/V^V^), POV‐alkoxide clusters. The higher energy feature corresponds to the V^IV^(d_xy_) → V^V^(d_x2‐y2_) intervalence charge transfer (IVCT), while the lower energy is assigned to a V^IV^(d_xy_) → V^V^(d_xy_) IVCT event.11c, 12a, 12d The intensity of the electronic transitions between V^IV^ and V^V^ centers vary with the number of V^IV^ and V^V^ within the clusters, thus giving insight into the oxidation state distribution of the metal centers. For example, if the reduction of **1‐[V_5_FeCl]** to **4‐K[V_5_FeCl]** is vanadium‐based, the molar absorptivity of the two IVCT bands should be halved, as the number of V^V^ ions decreases from two to one; we note that this prediction is observed. Furthermore, the absorbances of the 382 and the 990 nm bands of the **3‐[V_5_FeCl]SbCl_6_** are similar to those of **1‐[V_5_FeCl]**. This is consistent with V^IV^_3_V^V^_2_Fe^III^ and V^IV^_2_V^V^_3_Fe^III^ assignments for **1‐[V_5_FeCl]** and **3‐[V_5_FeCl]SbCl_6_**, respectively, as there are two pairs of V^IV^ and V^V^ ions for charge transfer to occur in both clusters.

In the case of complex **5‐(CoCp_2_)_2_[V_5_FeCl]**, the absence of the characteristic V^IV^ → V^V^ IVCT in the electronic absorption spectrum indicates an isovalent oxidation state distribution of vanadium ions within the cluster, that is, [V^IV^_5_Fe^III^]. In line with this oxidation state assignment, a low intensity absorption feature at 598 nm (235 cm^−1^ m ^−1^) is observed. Similar absorption features have been observed in other hexavanadate and heterometallic POV‐alkoxides, where all the vanadium ions are in the tetravalent state, and has been assigned as a transition localized on a single V^IV^ center.11b, 11c, 12b, 12c

An additional intense feature between 294 and 316 nm is observed for all redox isomers. In our previous work with heterometal‐functionalized POV‐alkoxide clusters bearing a chloride ligand (**1‐[V_5_FeCl]** and [V_5_O_6_(OCH_3_)_12_TiCl]), this has been assigned as a Cl:→M ligand‐to‐metal charge transfer (LMCT) event.12d, 14 The presence of this feature in each redox isomer confirms that the chloride ion remains bound to the cluster in solution. Notably, as the halide‐functionalized FePOV clusters are reduced, a hypsochromic shift in the Cl:→Fe^III^ transition occurs. This implies that while the redox activity of **1‐[V_5_FeCl]** is localized to the vanadium ions, the surrounding POV‐alkoxide framework affects the electronic structure of the Fe‐Cl moiety. Specifically, as the metalloligand becomes more electron rich, the ability of the Fe^III^ center to accept electron density from the chloride anion decreases. While oxygen‐to‐vanadium LMCT bands might also occur in these high‐energy regimes, the absence of these bands in the spectra of POV‐alkoxide clusters lacking halide ligands suggests that this feature indeed derives from an electronic transition from Cl to the heteroion (Figure S20).

Taken together, the spectroscopic data of complexes **1‐[V_5_FeCl]**, **3‐[V_5_FeCl]SbCl_6_**, **4‐K[V_5_FeCl]**, and **5‐(CoCp_2_)_2_[V_5_FeCl]**, confirm that the redox activity of the cluster is localized within the vanadate cluster core. This is consistent with our previous reports investigating the redox chemistry of heterometal‐functionalized POV‐alkoxide clusters.12b, 12c, 12d We can thus assign the oxidation states distributions of transition metal ions in the chloride‐functionalized, iron‐functionalized POV clusters as follows: **5‐(CoCp)_2_[V_5_FeCl]**=[V^IV^_5_Fe^III^], **4‐K[V_5_FeCl]**=[V^IV^_4_V^V^Fe^III^], **1‐[V_5_FeCl]**=[V^IV^_3_V^V^_2_Fe^III^], and **3‐[V_5_FeCl]SbCl_6_**=[V^IV^_2_V^V^_3_Fe^III^]. These assignments allow for a more appropriate comparison of the electronic structures of complex **1‐[V_5_FeCl]** and **2‐[V_5_FeX]** (X=OTf, ClO_4_) (*vide infra*).

### Theoretical analysis of the electrochemical properties of 1‐[V_5_FeCl] versus 2‐[V_5_Fe]ClO_4_

With the oxidation state distributions for the redox isomers of **1‐[V_5_FeCl]** identified, we became interested in gaining a fundamental understanding of how the coordination environment around the iron center imparts the different electrochemical behaviors observed in the CV of **1‐[V_5_FeCl]** and **2‐[V_5_Fe]ClO_4_**. As such, theoretical calculations were employed. First, the redox transitions of **1‐[V_5_FeCl]** (CN=6) and **2‐[V_5_Fe]ClO_4_** (CN=5) were obtained by DFT‐level calculations using the B3LYP functional *via* the Jaguar program suite (see SI for details).^15^ As shown in Table 1, the theoretically calculated reduction potentials for processes **1’**, **2’**, and **3’** for **1‐[V_5_FeCl]** are in excellent agreement with the experimentally observed values, with maximum deviations <0.1 V. However, for process **4’**, the experimental data (−0.68 V) differs significantly from our calculated value (−0.37 V). This is possibly related to solvation effects, where the acetonitrile solvation shell surrounding **1‐[V_5_FeCl]** is bound rather strongly as the negative charge is increased.^8^ For **2‐[V_5_Fe]ClO_4_**, where we expect dissociation of the perchlorate ion in solution, the calculations were performed for the 5‐coordinate iron center. The reduction potentials for the experimentally observed processes **2’**, **3’**, and **4’** are in excellent agreement with the calculated values, with maximum deviations <0.1 V. However, for process **5’**, we note a significant difference in calculated and experimental reduction potentials (Δ*E* ca. 1.7 V). This deviation is currently not understood and still under investigation. We hypothesize that it might be related to changes of the coordination environment around the iron center upon accessing the di‐anionic species.

**Table 1:** Experimental and calculated reduction potentials for **1‐[V_5_FeCl]** and **2‐[V_5_Fe]ClO_4_**. All potentials referenced against Fc^0/+^.

Redox process	V^IV^V^V^_4_Fe^III^/V^IV^_2_V^V^_3_Fe^III^		V^IV^_2_V^V^_3_Fe^III^/V^IV^_3_V^V^_2_Fe^III^		V^IV^_3_V^V^_2_Fe^III^/V^IV^_4_V^V^Fe^III^		V^IV^_4_V^V^Fe^III^/V^IV^_5_Fe^III^		V^IV^_5_Fe^III^/V^III^V^IV^_4_Fe^III^	
Step Number	**1’**		**2’**		**3’**		**4’**		**5’**	
	*E*_exp._/V	E_calcd_/V	*E*_exp._/V	E_calcd_/V	*E*_exp._/V	E_calcd_/V	*E*_exp._/V	E_calcd_/V	*E*_exp._/V	E_calcd_/V
**1‐[V_5_FeCl] (CN=6)**	0.83	0.83	0.31	0.34	−0.21	−0.28	−0.68	−0.37	—	—
**2‐[V_5_Fe]ClO_4_ (CN=5)**	—	—	0.21	0.21	−0.36	−0.33	−0.94	−0.85	−1.46	−3.23

**Figure 4:**
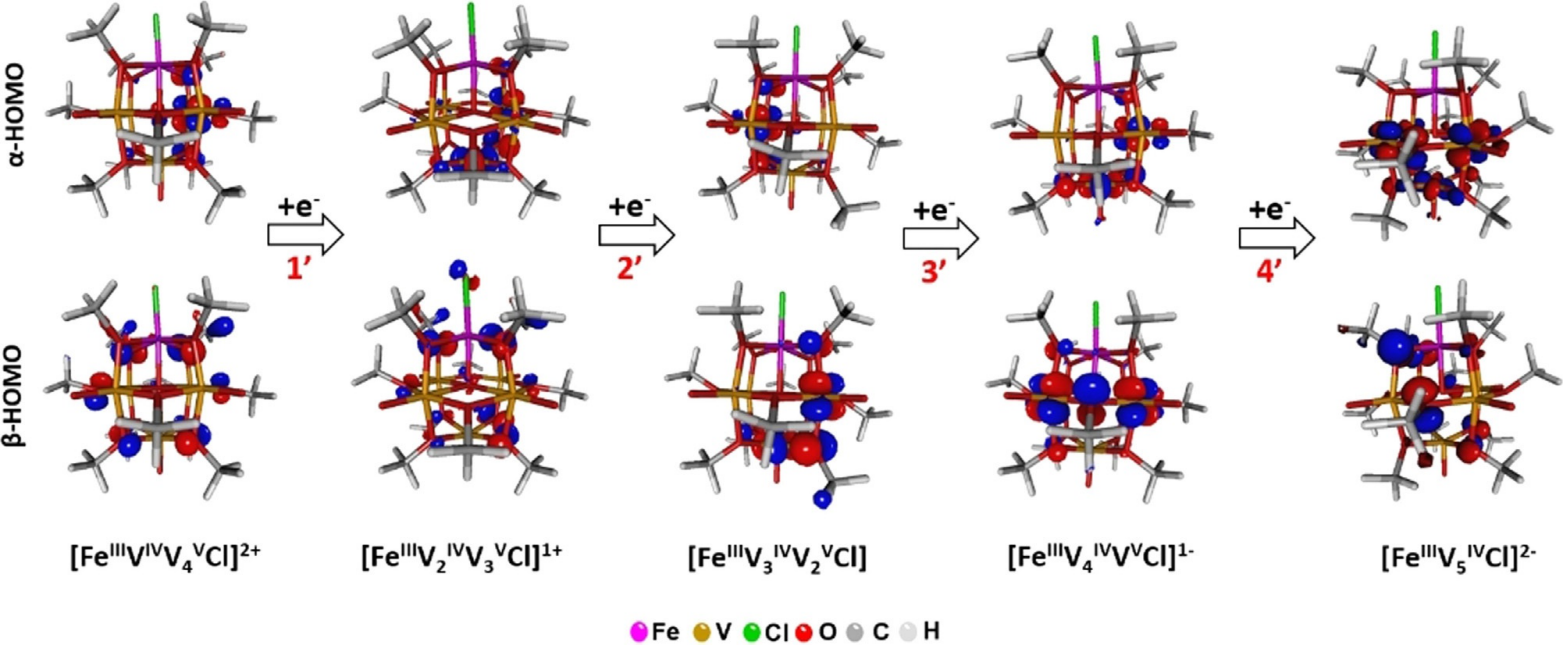
Frontier molecular orbital plots showing the highest occupied molecular orbitals (HOMO) for the complete set of redox‐isomers of **1‐[V_5_FeCl]**. Red numbers mark the reduction steps experimentally observed in cyclic voltammetry (Table 1).

Frontier molecular orbital analysis of the highest occupied molecular orbitals (HOMO) of all five possible redox isomers observed in the CV of **1‐[V_5_FeCl]** (Figure 4) indicate that all reduction processes **1’**, **2’**, **3’**, and **4’** lead to the reduction of the vanadium centers in **1‐[V_5_FeCl]**. This results in a retention of the ferric center throughout the series of redox isomers. Similar observations were made upon analysis of complex **2‐[V_5_Fe]ClO_4_** (Figure S21), where the calculation showed the redox activity to be localized to the POV scaffold. Furthermore, the atomic spin densities from Mulliken analysis for the complete set of redox‐isomers for both clusters shows that iron center remains as high‐spin iron(III) ion *S*_Fe_=^5^/_2_ (Table S5, S6). These assignments are consistent with the experimental results described above.

**Figure 5:**
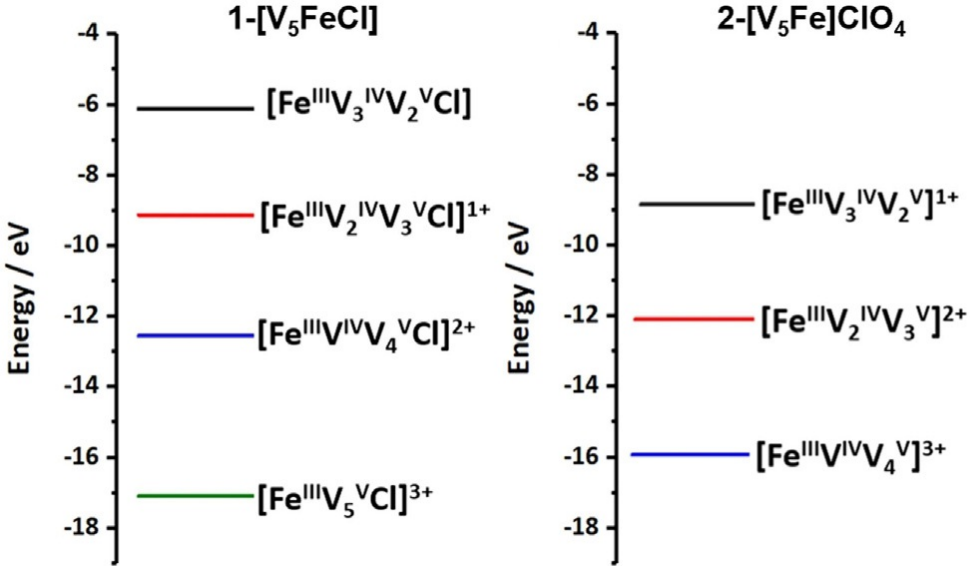
HOMO energies of **1‐[V_5_FeCl]** and **2‐[V_5_Fe]ClO_4_** upon oxidation, showing that the oxidation of **1‐[V_5_FeCl]** is energetically less demanding compared with **2‐[V_5_Fe]ClO_4_**.

With the validity of these calculations established, the individual redox profiles of **1‐[V_5_FeCl]** and **2‐[V_5_Fe]ClO_4_** were analyzed theoretically by DFT‐level computations to gain insights into the different behavior observed upon oxidation from the [Fe^III^V^IV^_3_V^V^_2_] form. These oxidative processes were selected to be interrogated since calculations were shown to model the E_1/2_ values for these processes better than their reductive counterparts. In particular, we calculated the HOMO energies of the clusters upon oxidation starting from the parent [V^IV^_3_V^V^_2_Fe^III^] state. As shown in Figure 5, we observe that for **1‐[V_5_FeCl]** the first and second oxidations are energetically more accessible when compared to the first and second oxidations of **2‐[V_5_Fe]ClO_4_**. This suggests that within the accessible solvent window, the first and second oxidation of **1‐[V_5_FeCl]** are energetically possible. In contrast, for **2‐[V_5_Fe]ClO_4_**, only the first oxidation is observed, while the second oxidation—while thermodynamically possible—is located outside the solvent stability window. These results are consistent with the increased negative charge provided by the chloride (or cyanate) anion stabilizing the oxidative processes.

### Rationalizing shifts in half‐wave potentials in transition metal functionalized POV‐alkoxide clusters

Comparing the CV of **1‐[V_5_FeCl]** to other previously reported heterometal‐functionalized POV‐alkoxide clusters (Figure S22, Table S7), the E_1/2_ values **1‐[V_5_FeCl]** and the gallium derivative are essentially identical. This is notable, as Ga^III^ has been used as a redox innocent surrogate for Fe^III^ in bioinorganic studies due to their similar charge and ionic radii.^16^ The redox profiles of these clusters are anodically shifted from the parent, hexavanadate cluster, [V_6_O_7_(OCH_3_)_12_], by about 0.10 V and cathodically shifted from the group 4 derivatives, [V_5_O_6_(OCH_3_)_12_M(OCH_3_)] (M=Ti^IV^, Zr^IV^, Hf^IV^), by about 0.40 V.

**Figure 6:**
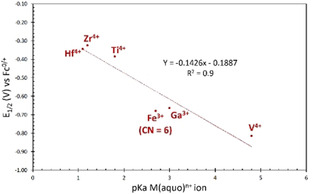
Plot of the half wave potential of the V^IV^_5_M/V^IV^_4_V^V^M redox couple (M=Hf^4+^, Zr^4+^, Ti^4+^, Fe^3+,^ Ga^3+^, and V^4+^) of POV‐alkoxide clusters (acetonitrile, 0.1 m [*n*Bu_4_N][PF_6_] as supporting electrolyte, referenced to Fc^0/+^) versus the pKa of the aqueous heteroion (M). CN refers to the coordination number around Fe^3+^. **2‐[V_5_Fe]ClO_4_** (CN=5) is excluded from this analysis.

In an effort to develop a model that rationalizes and predicts the observed shifts in the CV of heterometallic POV‐alkoxide clusters, we looked for correlations between quantitative parameters describing physical properties of the heteroions and the E_1/2_ values of the POV‐alkoxide compounds. Lewis acidity has been shown to be a good predictor for shifts in the electrochemical profiles of bimetallic^17^ and multi‐metallic^18^ systems. Specifically, the aqueous p*K*_a_, p*K*_a_(H_2_O), of the installed heteroion has been used as a measure of the Lewis acidity in order to quantify these effects. Taking inspiration from these studies, the electrochemical potentials of the [V^IV^_5_M]/[V^IV^_4_V^V^M] redox event for [V_5_O_6_(OCH_3_)_12_MX] (M=Hf^4+^, Zr^4+^, Ti^4+^, X=OCH_3_^−^; M=Fe^3+^, Ga^3+^, X=Cl^−^; M=V^4+^, X=O^2−^) clusters, in acetonitrile, were plotted against the p*K*_a_(M(OH_2_)_*n*_) of the installed heteroion (Figure 6, Table S7). Similar plots comparing E_1/2_ vs. p*K*_a_ (M(OH_2_)_*n*_) for the other redox events were analyzed and are reported in the supporting information file (Figure S23, Table S7). Good correlations between the positions of the vanadium‐based redox events and the Lewis acidity of the heteroion were observed (R^2^=0.79–0.90). The E_1/2_ values decreased by an average of −150±9 mV per p*K*_a_ unit, which is consistent with our previous analysis of redox dependence on Lewis acidity for these Lindqvist POV systems.12d Interestingly, the steepness of the slope steadily increases from about −140 mV/ p*K*_a_ for the most cathodic [V^IV^_5_M]/[V^IV^_4_V^V^M] couple to −160 mV/ p*K*_a_ for the most anodic [V^IV^_2_V^V^_3_M]/[V^IV^V^V^_4_M] couple. This suggests that the Lewis acidity of the heteroion has a greater influence on the redox properties of the more oxidized species of these clusters. Furthermore, these slopes are significantly steeper than those reported previously for heterometallic systems, specifically bimetallic, Schiff base cobalt (ca. −50 mV/p*K*_a_)17a and nickel (ca. −70 mV/ p*K*_a_)17c complexes and tetranuclear iron oxide (ca. −70 mV/p*K*_a_)18d and manganese oxide (ca. −100 mV/ p*K*_a_)18b, 18c clusters. Thus, for the heterometal‐functionalized POV‐alkoxide clusters, the electrochemistry of the surrounding vanadium centers appears to be particularly sensitive to the Lewis acceptor capability of the incorporated heteroion.

Notably, iron‐functionalized POV‐alkoxides with weakly coordinating anions, namely **2‐[V_5_Fe]ClO_4_** and **2‐[V_5_Fe]OTf**, deviate substantially from the linear relationship between the Lewis acidity of the installed heteroion established for all other heterometallic, POV‐alkoxide clusters reported to date (Figure S24, Table S7).12d For example, **2‐[V_5_Fe]ClO_4_** exhibits E_1/2_ values at significantly more reducing potentials (shifted by ca. −0.17 V) compared to that of **1‐[V_5_FeCl]**. This is likely due to the fact that the aqua species used for these pK_a_ determinations are six‐coordinate iron centers, thus rendering these values poor representations of the Lewis acidity of the 5‐coordinate iron center.

The differences in the electronic profiles of the 5‐coordinate and 6‐coordinate iron‐functionalized clusters run deeper than the overall shift in the redox profiles of the two clusters. For example, complex **2‐[V_5_Fe]ClO_4_** supports an additional cathodic event at −1.46 V, which was assigned to a [V^IV^_5_Fe^III^]/[V^III^V^IV^_4_Fe^III^] couple, that does not occur in **1‐[V_5_FeCl]**. These results are counter‐intuitive, given that, based on the cathodic shift in the CV, **2‐[V_5_Fe]ClO_4_** is clearly more electron rich as compared to its 6‐coordinate congener. Furthermore, only one oxidized species of **2‐[V_5_Fe]ClO_4_** ([V^IV^_2_V^V^_3_Fe^III^]^2+^) has been observed experimentally, while the CV of **1‐[V_5_FeCl]** displayed two oxidation processes ([V^IV^_3_V^V^_2_Fe^III^]/[V^IV^_2_V^V^_3_Fe^III^]^1+^ and [V^IV^_2_V^V^_3_Fe^III^]^1+^/[V^IV^V^V^_4_Fe^III^]^2+^). Overall, the ligand environment surrounding the iron influences the electrochemical behavior of these iron‐functionalized clusters in a complex manner such that simple electrostatic and Lewis acidity arguments cannot predict. In the cobalt‐doped polyoxotitanate cages, [Ti_4_O(OC_2_H_5_)_15_CoX] and [Ti_7_O_5_(OC_2_H_5_)_19_CoX] (X = OC_2_H_5_^‐^, F^‐^, Cl^‐^, Br^‐^, and I^‐^), the ligand field of cobalt center has a significant effect on the optical band gap of these clusters.^19^ Similarly, ligand field effects could also be significant for the open‐shell Fe^III^ center in the iron‐functionalized, POV‐alkoxides reported herein. We still do not fully understand the cause for the differences in the electrochemistry of **1‐[V_5_FeCl]** and **2‐[V_5_Fe]ClO_4_**, which is the subject of ongoing investigations by our research team.

## Conclusions

Herein, combined experimental and theoretical analyses of the electrochemical features of two iron‐polyoxovanadate‐alkoxide clusters have been studied, focusing on the changes in electrochemical behavior between five‐coordinate and six‐coordinate iron sites. Coordination of a chloride to the iron(III) center led to an anodic shift in the redox events and stabilization of an additional oxidative feature in the CV. Both experimental and theoretical studies showed that storage and release of electron density is localized within the vanadate cluster core for **1‐[V_5_FeCl]**, which was also seen in previously reported **2‐[V_5_Fe]ClO_4_**. Furthermore, changing the geometry of the ferric center from square pyramidal to octahedral upon coordination of the chloride ligand appears to tune these vanadium‐based redox events by adjusting the orbital overlap between the iron and the POV metalloligand. Therefore, both the type and the ligand environment of the installed heteroion will be important in selectively designing polyoxovanadate clusters with specific electrochemical properties.

## Experimental Section

**General Considerations**: All manipulations were carried out in the absence of water and oxygen in a UniLab MBraun inert atmosphere glovebox under an atmosphere of dinitrogen. Glassware was oven dried for a minimum of 4 hours and cooled in an evacuated antechamber prior to use in the drybox. Celite 545 (J. T. Baker) was dried in a Schlenk flask for at least 14 hours at 150 °C under vacuum prior to use. 3 Å molecular sieves (Fisher Scientific) were activated using the same drying method. All solvents were dried and deoxygenated on a Glass Contour System (Pure Process Technology, LLC) and stored over activated 3 Å molecular sieves. Nitrosonium hexafluorophosphate (NOPF_6_, 96 %) was purchased from Alfa Aesar and used as received. Tris(4‐bromophenyl)ammoniumyl hexachloroantimonate ((N(C_6_H_4_Br‐4)_3_)SbCl_6_, technical grade) and bis(cyclopentadienyl)cobalt(II) (CoCp_2_, 98 %) were purchased from Sigma–Aldrich and used as received. Tetrabutylammonium hexafluorophosphate ((*n*Bu_4_N)PF_6_, 98 %) was also purchased from Sigma–Aldrich, recrystallized three times from hot ethanol, and stored under dynamic vacuum in the glovebox prior to use. Potassium graphite (KC_8_), [V_5_O_6_(OCH_3_)_12_FeCl] (**1‐[V_5_FeCl]**), [V_5_O_6_(OCH_3_)_12_Fe]ClO_4_ (**2‐[V_5_Fe]ClO_4_**), and [V_5_O_6_(OCH_3_)_12_FeOCN] (**2‐[V_5_FeOCN]**)were prepared according to literature precedent.12b, 12d, 20

^1^H NMR spectra were recorded on a Bruker DPX‐400 MHz spectrometer locked on the signal of deuterated solvents. All chemical shifts were reported relative to the peak of residual ^1^H signal in the deuterated solvents. CD_3_CN and [D_8_]THF were purchased from Cambridge Isotope Laboratories, degassed by three freeze‐pump‐thaw cycles, and stored over activated 3 Å molecular sieves. Infrared (FT‐IR, ATR) spectra of complexes were recorded on a Shimadzu IRAffinity‐1 Fourier Transform Infrared spectrophotometer and were reported in wavenumbers (cm^−1^). Electronic absorption measurements were recorded at room temperature in anhydrous acetonitrile in a sealed 1 cm quartz cuvette with an Agilent Cary 60 UV/Vis spectrophotometer. A single crystal of **4‐CoCp_2_[V_5_FeCl]**, was placed on the tip of a thin glass optical fiber (goniometer head) and mounted on a Rigaku XtaLab Synergy‐S Dualflex diffractometer equipped with a HyPix‐6000HE HPC area detector for data collection at 100.00(10) K. The structure was solved using ShelXT and refined using ShelXL. Elemental analyses were performed on a PerkinElmer 2400 Series II Analyzer at the CENTC Elemental Analysis Facility, University of Rochester.

Cyclic Voltammetry measurements were carried out at room temperature in a nitrogen filled glove box, using a Bio‐Logic SP 150 potentiostat/galvanostat and the EC‐lab software suite. Cyclic voltammograms were recorded using a glassy carbon working electrode (ø=3.0 mm) and a Pt wire auxiliary electrode, both purchased from CH Instruments, USA. An Ag/Ag^+^ non‐aqueous reference electrode with 0.01 m AgNO_3_ in 0.05 m (*n*Bu_4_N)PF_6_ in CH_3_CN was purchased from Bio‐Logic and used as the reference electrode for all cyclic voltammetry measurements. The cyclic voltammograms were collected with 1 mm active species in 0.1 m ([*n*Bu_4_N)PF_6_ solutions in dry acetonitrile. All redox events were referenced against Fc^+/0^ redox couple.

Synthesis of [V_5_O_6_(OCH_3_)_12_FeCl]SbCl_6_⋅(THF)_0.5_ (**3‐[V_5_FeCl]SbCl_6_**): In a glovebox, a 20 mL scintillation vial was charged with **1‐[V_5_FeCl]** (97 mg, 0.12 mmol, 1.0 equiv) and 10 mL acetonitrile. Solid (N(C_6_H_4_Br‐4)_3_)SbCl_6_ (95 mg, 0.12 mmol, 1.0 equiv) was then added in small portions. The cloudy, dark green mixture was stirred at room temperature for thirty minutes. The reaction mixture was filtered over a bed of Celite (1.0 cm) on a medium‐porosity frit, and the volatiles were removed under reduced pressure. The resulting dark green solid was triturated with toluene (10 mL *x* 5) and diethyl ether (10 mL). The solid was extracted with acetonitrile (2 mL *x* 4) and filtered over a bed of Celite (1.0 cm) on a medium‐porosity frit. The dark green solution was concentrated to half the original volume and was allowed to crystallize at −30 °C overnight. Complex **3‐[V_5_FeCl]SbCl_6_** was isolated as a shiny, green solid (48 mg, 0.04 mmol, 35 % yield based on **1‐[V_5_FeCl]**). ^1^H NMR (400 MHz, CD_3_CN): *δ*=14.59 (fwhh=260 Hz), 12.71 (fwhh=400 Hz). FT‐IR (ATR, cm^−1^): 984 (*V=*O_t_). UV/Vis [CH_3_CN; λ, nm (*ϵ*, 1×10^3^ m ^−1^ cm^−1^)]: 316 (9.92), 382 (8.01), 992 (0.66). Elemental analysis calculated for C_12_H_36_O_18_V_5_FeSbCl_7_^.^ 1/2 THF (%) (MW=1184.92 g mol^−1^): C, 14.19; H, 3.06; Found (%): C, 14.39; H, 2.83.

Synthesis of K[V_5_O_6_(OCH_3_)_12_FeCl]⋅(MeCN)_1.25_(THF)_0.25_ (**4‐K[V_5_FeCl]**): In a glovebox, a 20 mL scintillation vial was charged with **1‐[V_5_FeCl]** (199 mg, 0.25 mmol, 1.0 equiv) and 18 mL tetrahydrofuran. Solid KC_8_ (32 mg, 0.24 mmol, 1.0 equiv) was then added in small portions. The cloudy, dark green mixture was stirred at room temperature for an hour. The reaction mixture was filtered over a bed of Celite (1.0 cm) on a medium‐porosity frit, and the volatiles were removed under reduced pressure. The resulting dark green solid was triturated with toluene (10 mL *x* 3), dichloromethane (10 mL *x* 3), and diethyl ether (10 mL *x* 3). The solid was extracted with acetonitrile, filtered over a bed of Celite (1.0 cm) on a medium‐porosity frit, and the volatiles were removed under reduced pressure, affording complex **4‐K[V_5_FeCl]** as a dark green solid (181 mg, 0.21 mmol, 87 % yield based on **1‐[V_5_FeCl]**). ^1^H NMR (400 MHz, CD_3_CN): *δ*=23.76 (fwhh=396 Hz), 18.09 (fwhh=620 Hz), 5.66 (fwhh=28 Hz). FT‐IR (ATR, cm^−1^): 1020 (O_b_‐CH_3_), 957 (*V=*O_t_). Elemental analysis calculated for C_12_H_36_O_18_V_5_FeKCl^.^ 11/4 MeCN 1/4 THF (%) (MW=992.85 g mol^−1^): C, 20.17; H, 4.56; N, 1.90; Found (%): C, 20.27; H, 4.44; N, 2.12.

Synthesis of CoCp_2_[V_5_O_6_(OCH_3_)_12_FeCl]⋅(DCM)_0.5_ (**4‐CoCp_2_[V_5_FeCl]**): In a glovebox, a 20 mL scintillation vial was charged with **1‐[V_5_FeCl]** (116 mg, 0.14 mmol, 1.0 equiv) and 12 mL tetrahydrofuran. Solid CoCp_2_ (32 mg, 0.17 mmol, 1.2 equiv) was then added in small portions. The cloudy, dark green mixture was stirred at room temperature for an hour. The reaction mixture was filtered over a bed of Celite (1.0 cm) on a medium‐porosity frit, and the volatiles were removed under reduced pressure. The resulting dark green solid was triturated with toluene (10 mL *x* 3) and diethyl ether (10 mL *x* 3). The solid was extracted with dichloromethane, filtered over a bed of Celite (1.0 cm) on a medium‐porosity frit, and the volatiles were removed under reduced pressure, affording complex **4‐CoCp_2_[V_5_FeCl]** as a dark green solid (103 mg, 0.10 mmol, 73 % yield based on **1‐[V_5_FeCl]**). Crystals suitable for X‐ray analysis were grown from slow diffusion of pentane into a saturated solution of **4‐CoCp_2_[V_5_FeCl]** in tetrahydrofuran. ^1^H NMR (400 MHz, CD_3_CN): *δ*=23.76 (fwhh=396 Hz), 18.09 (fwhh=620 Hz), 5.66 (fwhh=28 Hz). FT‐IR (ATR, cm^−1^): 1024 (O_b_‐CH_3_), 959 (*V=*O_t_). UV/Vis [CH_3_CN; λ, nm (*ϵ*, 1×10^3^ m ^−1^ cm^−1^)]: 300 (9.71), 382 (3.65), 992 (0.55). Elemental analysis calculated for C_22_H_46_O_18_V_5_FeCoCl^.^ 1/2 DCM (%) (MW=1045.99 g mol^−1^): C, 25.84; H, 4.53; Found (%): C, 25.89; H, 4.23.

Synthesis of (CoCp_2_)_2_[V_5_O_6_(OCH_3_)_12_FeCl]⋅(DCM)_0.5_ (**5‐(CoCp_2_)_2_[V_5_FeCl]**): In a glovebox, a 20 mL scintillation vial was charged with **1‐[V_5_FeCl]** (143 mg, 0.18 mmol, 1.0 equiv) and 16 mL tetrahydrofuran. Solid CoCp_2_ (66 mg, 0.35 mmol, 2.0 equiv) was then added in small portions. The teal slurry was stirred at room temperature for an hour. The precipitate was collected over a bed of Celite (1.0 cm) on a medium‐porosity frit and triturated with fresh tetrahydrofuran (10 mL *x* 3). The remaining teal solid was extracted with acetonitrile (2 mL *x* 3), and the volatiles were removed under reduced pressure. The resulting solid was triturated with dichloromethane (10 mL *x* 3), affording complex **5‐(CoCp_2_)_2_[V_5_FeCl]** as a teal solid (88 mg, 0.07 mmol, 42 % yield based on **1‐[V_5_FeCl]**). Crystals suitable for X‐ray analysis were grown from slow diffusion of diethyl ether into a saturated solution of **5‐(CoCp_2_)_2_[V_5_FeCl]** in dichloromethane. ^1^H NMR (400 MHz, CD_3_CN): *δ*=24.91, 21.88, 5.69 (92 Hz). FT‐IR (ATR, cm^−1^): 1043 (O_b_‐CH_3_), 941 (*V=*O_t_). UV/Vis [CH_3_CN; λ, nm (*ϵ*, 1×10^3^ m ^−1^ cm^−1^)]: 296 (7.16), 598 (0.24). Elemental analysis calculated for C_32_H_56_O_18_V_5_FeCo_2_Cl ^.^ 1/2 DCM (%) (MW=1235.12 g mol^−1^): C, 31.61; H, 4.65; Found (%): C, 31.62; H, 4.38.

## Conflict of interest

The authors declare no conflict of interest.

## Biographical Information

*Carsten Streb is Director of the Institute of Inorganic Chemistry I at Ulm University and group leader at the Helmholtz Institute Ulm. His love for POMs started during his PhD, working with Lee Cronin at the University of Glasgow. His current research is focused on designing POM‐based functional materials and composites to address global chemical challenges including energy conversion/storage, water purification and public health. In particular, he enjoys exploring the supramolecular chemistry and metal‐functionalization of molecular vanadium oxide clusters*.

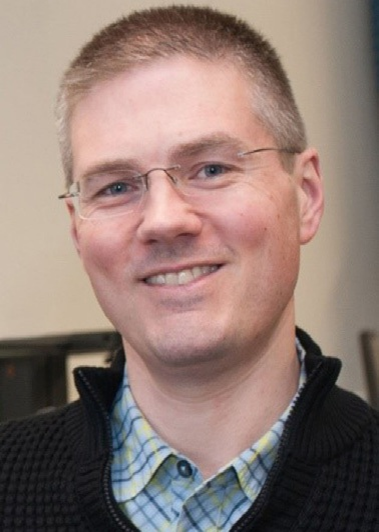

## Biographical Information

*Montaha Anjass is independent research group leader and Margarete‐von‐Wrangell‐Fellow at Ulm University and Helmholtz Institute Ulm. She received her B.Sc. degree in Chemistry from Birzeit University, Palestine and her M.Sc. degree in Advanced Materials from Ulm University, Germany. In 2019, she completed her PhD (supervisors: C. Streb, M. Fichtner, T. Jacob) at Ulm University. From 2019–2020 she undertook a postdoc at Helmholtz‐Institute Ulm. Her current research interests are advanced battery materials based on redox‐active molecular metal oxides and electrically conductive organic polymers and their use in (post‐)lithium batteries*.

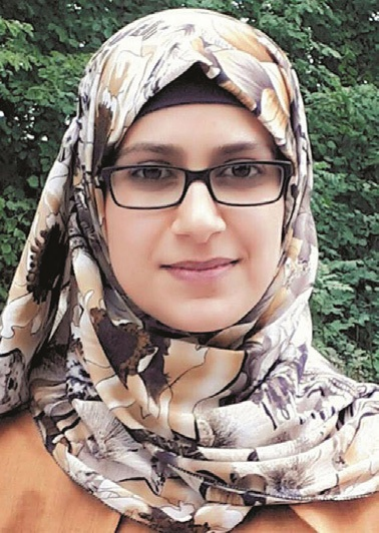

## Biographical Information

*Ellen Matson received her PhD from Purdue University. Following a postdoctoral appointment at the University of Illinois at Urbana‐Champaign, Matson began her independent career at the University of Rochester (2015), where she is currently the Wilmot Assistant Professor of Chemistry. The Matson Laboratory studies the synthesis and reactivity of reduced vanadium oxide clusters. Ellen Matson has earned several awards recognizing early success in research and teaching, including a Sloan Fellowship, a Cottrell Scholar Award, a Camille Dreyfus Teaching‐Scholar Award and the Edith Flanigen Award*.

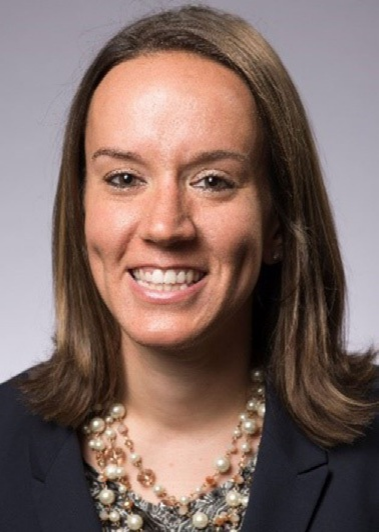

## Supporting information

As a service to our authors and readers, this journal provides supporting information supplied by the authors. Such materials are peer reviewed and may be re‐organized for online delivery, but are not copy‐edited or typeset. Technical support issues arising from supporting information (other than missing files) should be addressed to the authors.

Supplementary

## References

[1a] S. Wang , W. Sun , Q. Hu , H. Yan , Y. Zeng , Bioorg. Med. Chem. Lett. 2017, 27, 2357–2359; 28431877 10.1016/j.bmcl.2017.04.025

[1b] D.-D. Zhang , Z.-Y. Guo , P.-F. Guo , X. Hu , X.-W. Chen , J.-H. Wang , ACS Appl. Mater. Interfaces 2018, 10, 21876–21882. 29882647 10.1021/acsami.8b05334

[2a] E. Coronado , C. J. Gómez-García , Chem. Rev. 1998, 98, 273–296; 11851506 10.1021/cr970471c

[2b] D.-L. Long , E. Burkholder , L. Cronin , Chem. Soc. Rev. 2007, 36, 105–121; 17173149 10.1039/b502666k

[2c] Y.-F. Song , R. Tsunashima , Chem. Soc. Rev. 2012, 41, 7384–7402; 22850732 10.1039/c2cs35143a

[2d] A. Proust , B. Matt , R. Villanneau , G. Guillemot , P. Gouzerh , G. Izzet , Chem. Soc. Rev. 2012, 41, 7605–7622. 22782306 10.1039/c2cs35119f

[3a] A. Müller , F. Peters , M. T. Pope , D. Gatteschi , Chem. Rev. 1998, 98, 239–272; 11851505 10.1021/cr9603946

[3b] J. M. Clemente-Juan , E. Coronado , A. Gaita-Ariño , Chem. Soc. Rev. 2012, 41, 7464–7478. 22948854 10.1039/c2cs35205b

[4a] X. Zhao , S. Zhang , J. Yan , L. Li , G. Wu , W. Shi , G. Yang , N. Guan , P. Cheng , Inorg. Chem. 2018, 57, 5030–5037; 29667839 10.1021/acs.inorgchem.8b00098

[4b] I. A. Weinstock , R. E. Schreiber , R. Neumann , Chem. Rev. 2018, 118, 2680–2717; 29192770 10.1021/acs.chemrev.7b00444

[4c] S.-S. Wang , G.-Y. Yang , Chem. Rev. 2015, 115, 4893–4962. 25965251 10.1021/cr500390v

[5a] M. Genovese , K. Lian , Curr. Opin. Solid St. Mater. Sci. 2015, 19, 126–137;

[5b] Y. Ji , L. Huang , J. Hu , C. Streb , Y.-F. Song , Energy Environ. Sci. 2015, 8, 776–789.

[6] M. Sadakane , E. Steckhan , Chem. Rev. 1998, 98, 219–238. 11851504 10.1021/cr960403a

[7a] H. D. Pratt , W. R. Pratt , X. Fang , N. S. Hudak , T. M. Anderson , Electrochim. Acta 2014, 138, 210–214;

[7b] H. D. Pratt , N. S. Hudak , X. Fang , T. M. Anderson , J. Power Sources 2013, 236, 259–264;

[7c] L. E. VanGelder , E. M. Matson , J. Mater. Chem. A 2018, 6, 13874–13882;

[7d] L. E. VanGelder , A. M. Kosswattaarachchi , P. L. Forrestel , T. R. Cook , E. M. Matson , Chem. Sci. 2018, 9, 1692–1699; 29675217 10.1039/c7sc05295b PMC5890794

[7e] L. E. VanGelder , B. E. Petel , O. Nachtigall , G. Martinez , W. W. Brennessel , E. M. Matson , ChemSusChem 2018, 11, 4139–4149. 30320959 10.1002/cssc.201802029

[8] J.-J. J. Chen , M. A. Barteau , Indust. Eng. Chem. Res. 2016, 55, 9857–9864.

[9] Y. Hayashi , Coord. Chem. Rev. 2011, 255, 2270–2280.

[10a] J. Tucher , L. C. Nye , I. Ivanovic-Burmazovic , A. Notarnicola , C. Streb , Chem. Eur. J. 2012, 18, 10949–10953; 22829485 10.1002/chem.201200404

[10b] J. Forster , B. Rösner , M. M. Khusniyarov , C. Streb , Chem. Commun. 2011, 47, 3114–3116; 10.1039/c0cc05536k 21258702

[10c] K. Kastner , J. Forster , H. Ida , G. N. Newton , H. Oshio , C. Streb , Chem. Eur. J. 2015, 21, 7686–7689; 25850969 10.1002/chem.201501049

[10d] C. Aronica , G. Chastanet , E. Zueva , S. A. Borshch , J. M. Clemente-Juan , D. Luneau , J. Am. Chem. Soc. 2008, 130, 2365–2371; 18215045 10.1021/ja078030q

[10e] J. M. Cameron , G. N. Newton , C. Busche , D.-L. Long , H. Oshio , L. Cronin , Chem. Commun. 2013, 49, 3395–3397; 10.1039/c3cc40912k 23511640

[10f] M. H. Anjass , K. Kastner , F. Nägele , M. Ringenberg , J. F. Boas , J. Zhang , A. M. Bond , T. Jacob , C. Streb , Angew. Chem. Int. Ed. 2017, 56, 14749–14752; 10.1002/anie.201706828 28906058

[11a] J. Spandl , C. Daniel , I. Brüdgam , H. Hartl , Angew. Chem. Int. Ed. 2003, 42, 1163–1166; 10.1002/anie.200390306 12640650

[11b] C. Daniel , H. Hartl , J. Am. Chem. Soc. 2005, 127, 13978–13987; 16201820 10.1021/ja052902b

[11c] C. Daniel , H. Hartl , J. Am. Chem. Soc. 2009, 131, 5101–5114. 19301901 10.1021/ja8073648

[12a] F. Li , L. E. VanGelder , W. W. Brennessel , E. M. Matson , Inorg. Chem. 2016, 55, 7332–7334; 27438070 10.1021/acs.inorgchem.6b01349

[12b] F. Li , S. H. Carpenter , R. F. Higgins , M. G. Hitt , W. W. Brennessel , M. G. Ferrier , S. K. Cary , J. S. Lezama-Pacheco , J. T. Wright , B. W. Stein , M. P. Shores , M. L. Neidig , S. A. Kozimor , E. M. Matson , Inorg. Chem. 2017, 56, 7065–7080; 28548499 10.1021/acs.inorgchem.7b00650

[12c] L. E. VanGelder , W. W. Brennessel , E. M. Matson , Dalton Trans. 2018, 47, 3698–3704; 29292450 10.1039/c7dt04455k

[12d] R. L. Meyer , W. W. Brennessel , E. M. Matson , Polyhedron 2018, 156, 303–311.

[13] N. G. Connelly , W. E. Geiger , Chem. Rev. 1996, 96, 877–910. 11848774 10.1021/cr940053x

[14] L. E. VanGelder , P. L. Forrestel , W. W. Brennessel , E. M. Matson , Chem. Commun. 2018, 54, 6839–6842. 10.1039/c8cc01517a 29700509

[15a] K. Yu. Monakhov , O. Linnenberg , P. Kozłowski , J. van Leusen , C. Besson , T. Secker , A. Ellern , X. López , J. M. Poblet , P. Kögerler , Chem. Eur. J. 2015, 21, 2387–2397; 25403795 10.1002/chem.201403858

[15b] S. Herrmann , N. Aydemir , F. Nägele , D. Fantauzzi , T. Jacob , J. Travas-Sejdic , C. Streb , Adv. Funct. Mater. 2017, 27, 1700881;

[15c] A. D. Bochevarov , E. Harder , T. F. Hughes , J. R. Greenwood , D. A. Braden , D. M. Philipp , D. Rinaldo , M. D. Halls , J. Zhang , R. A. Friesner , Int. J. Quant. Chem. 2013, 113, 2110–2142.

[16a] Y. Kaneko , M. Thoendel , O. Olakanmi , B. E. Britigan , P. K. Singh , J. Clin. Invest. 2007, 117, 877–888; 17364024 10.1172/JCI30783 PMC1810576

[16b] C. R. Chitambar , M. M. Al-Gizawiy , H. S. Alhajala , K. R. Pechman , J. P. Wereley , R. Wujek , P. A. Clark , J. S. Kuo , W. E. Antholine , K. M. Schmainda , Mol. Cancer Therap. 2018, 17, 1240–1250; 29592883 10.1158/1535-7163.MCT-17-1009 PMC5984712

[16c] C. R. Chitambar , Pharmacological Research 2017, 115, 56–64; 27856328 10.1016/j.phrs.2016.11.009

[16d] C. R. Chitambar , Biochim. Biophys. Acta Mol. Cell Res. 2016, 1863, 2044–2053; 10.1016/j.bbamcr.2016.04.027 27150508

[16e] R. Shannon , Acta Crystallogr. Sect. A 1976, 32, 751–767.

[17a] A. H. Reath , J. W. Ziller , C. Tsay , A. J. Ryan , J. Y. Yang , Inorg. Chem. 2017, 56, 3713–3718; 28240885 10.1021/acs.inorgchem.6b03098

[17b] T. Chantarojsiri , J. W. Ziller , J. Y. Yang , Chem. Sci. 2018, 9, 2567–2574; 29732136 10.1039/c7sc04486k PMC5911827

[17c] A. Kumar , D. Lionetti , V. W. Day , J. D. Blakemore , Chem. Eur. J. 2018, 24, 141–149. 29024095 10.1002/chem.201704006

[18a] V. Krewald , F. Neese , D. A. Pantazis , Phys. Chem. Chem. Phys. 2016, 18, 10739–10750; 26762578 10.1039/c5cp07213a

[18b] E. Y. Tsui , T. Agapie , Proc. Natl. Acad. Sci. USA 2013, 110, 10084–10088; 23744039 10.1073/pnas.1302677110 PMC3690856

[18c] E. Y. Tsui , R. Tran , J. Yano , T. Agapie , Nat. Chem. 2013, 5, 293–299; 23511417 10.1038/nchem.1578 PMC3654670

[18d] D. E. Herbert , D. Lionetti , J. Rittle , T. Agapie , J. Am. Chem. Soc. 2013, 135, 19075–19078; 24304416 10.1021/ja4104974 PMC3953215

[18e] P.-H. Lin , M. K. Takase , T. Agapie , Inorg. Chem. 2015, 54, 59–64. 25521310 10.1021/ic5015219 PMC4286168

[19] S. Hanf , P. D. Matthews , N. Lo , H.-K. Luo , D. S. Wright , Dalton Trans. 2017, 46, 578–585. 27981324 10.1039/c6dt04288k

[20] I. S. Weitz , M. Rabinovitz , J. Chem. Soc. Perkin Trans. 1993, 117–120.

