# Supplementary material for: Electronic Consequences of Ligand Substitution at Heterometal Centers in Polyoxovanadium Clusters: Controlling the Redox Properties through Heterometal Coordination Number
Source: Chemistry. 2020 Jun 25;26(44):9905–14. doi: 10.1002/chem.201905624 (PMC7496301; doi:10.1002/chem.201905624)
Supplement: Supplementary file 1 — Supplementary [file CHEM-26-9905-s001.pdf]

Chemistry–A European Journal

Supporting Information

Electronic Consequences of Ligand Substitution at Heterometal Centers in Polyoxovanadium Clusters: Controlling the Redox Properties through Heterometal Coordination Number

Rachel L. Meyer,[a] Montaha H. Anjass,*[b, c] Brittney E. Petel,[a] William W. Brennessel,[a] Carsten Streb,*[b, c] and Ellen M. Matson*[a]

Supporting Information (SI)

Electrochemical Consequences of Ligand Substitution at Heterometal Centers in Polyoxovanadium Clusters: Controlling the Redox Properties *via* Heterometal Coordination Number

Rachel L. Meyer,[a] Montaha H. Anjass,*[b,c] Brittney E. Petel,[a] William W. Brennessel,[a] Carsten Streb,*[b,c] and Ellen M. Matson*[a]

[a] R. L. Meyer, B. E. Petel, Dr. W. W. Brennessel and Prof. E. M. Matson
Department of Chemistry
University of Rochester
Rochester NY 14627 ISA
E-mail: matson@chem.rochester.edu

[b] Dr. M. H. Anjass and Prof. C. Streb
Institute of Inorganic Chemistry I
Ulm University
Albert-Einstein-Allee 11 D-89081, Ulm, Germany
E-mail: montaha.anjass@uni-ulm.de
E-mail: carsten.streb@uni-ulm.de

[c] Dr. M. H. Anjass and Prof. C. Streb
Helmholtz Institute Ulm (HIU)
Helmholtzstrasse 11, D-89081 Ulm, Germany

Supporting Information Table of Contents:

General Considerations of Titration Study and Theoretical Analysis ……………………………..S3-S4

Figure S1. CV of **1-[V_5FeCl]** from 2 to -2 V ……………………………………………………..……....S4

Figure S2. CV of **1-[V_5FeCl]**, **2-[V_5Fe]ClO_4**, and **1-[V_5FeOCN]** ……….…………………………….......S4

Table S1. $E_{1/2}$ values for **1-[V_5FeCl]**, **2-[V_5Fe]ClO_4**, and **1-[V_5FeOCN]** ………………………….…S5

Figure S3. CV of **1-[V_5FeOCN]** from 2 to -2 V ……………….…………………………..…..………......S5

Figure S4. ^{1}H NMR spectrum of **3-[V_5FeCl]$SbCl_6$** in CD_3CN ……………………………………..……....S6

Figure S5. ^{1}H NMR spectrum of **3-[V_5FeCl]$SbCl_6$** in THF-d_8 …………...…………………………......S6

Figure S6. ESI-MS (+*ve*) of **3-[V_5FeCl]$SbCl_6$** …………..…………………………….……………......S7

Figure S7. ^{1}H NMR and infrared spectra **1-[V_5FeCl]** + 2 equiv. $NOPF_6$ …………………………………S8

Figure S8. Bulk oxidation of **1-[V_5FeCl]** …..…….……………………..………………………….………..….S9

Figure S9. CV of +0.85 V event of **1-[V_5FeCl]** at varying scan rates ……..……………………….……..…..S10

Figure S10. CV of **1-[V_5FeCl]** titrated with (^{n}Bu$_4$N)Cl …………………..……………….……....…..S11

Figure S11. ^{1}H NMR spectrum of **4-K[V_5FeCl]** ….………………...……………..…….……………....S12

Figure S12. ESI-MS (-*ve*) of **4-K[V_5FeCl]** ….…………………….…...….………………….…………...S13

Figure S13. ^{1}H NMR and infrared spectra of **1-[V_5FeCl]** + 2 equiv. KC_8 ….…………………………....S14

Figure S14. ^{1}H NMR spectrum of **5-($CoCp_2$)$_2$[V_5FeCl]** ………………………....……….,…………..S15

Figure S15. ESI-MS (-*ve*) of **5-($CoCp_2$)$_2$[V_5FeCl]** ……………………………....……….,………..…S16

Figure S16. Molecular structure of **5-($CoCp_2$)$_2$[V_5FeCl]** …..…………………………..…..……...….S17

Figure S17. ^{1}H NMR spectrum of **4-$CoCp_2$[V_5FeCl]** …..…………………………..……………..…S18

Figure S18. ESI-MS (-*ve*) of **4-$CoCp_2$[V_5FeCl]** …..…………………………...……..………………S19

Figure S19. Molecular structure of **4-$CoCp_2$[V_5FeCl]** …..………………………...…..……...……..S20

Table S2. Crystallographic parameters of **4-$CoCp_2$[V_5FeCl]** and **5-($CoCp_2$)$_2$[V_5FeCl]** ………………S20

Table S3. Values for key FT-IR bands for the redox isomers of **1-[V_5FeCl]** ……………….…………S21

Table S4. Values for electronic absorption features for the redox isomers of **1-[V_5FeCl]** ……….………S21

Figure S20. Electronic absorption spectra of POV-alkoxide clusters…………………………....……...S21

Figure S21. Calculated frontier molecular orbitals for the redox isomers of **2-[V_5Fe]ClO_4**……..……...S22

Figure S22. Cyclic voltammograms of heterometal-functionalized POV-alkoxide clusters……….…...S22

Table S5. pKa and coordination number of heteroion and $E_{1/2}$ values of POV-alkoxide clusters………...S23

Figure S23. Plots of $E_{1/2}$ vs. pKa for POV-alkoxide clusters, excluding **2-[V_5Fe]ClO_4** ..……….….……S23

Figure S24. Plots of $E_{1/2}$ vs. pKa for POV-alkoxide clusters, including **2-[V_5Fe]ClO_4** ..……….………S24

References……………………………...…………………………………………………...…S24

Experimental details for titration of 1-[V_5FeCl] with [^{n}Bu_4N]Cl: A 5 mL acetonitrile solution of **1-[V_5FeCl]** (1.7 mM) with [^{n}Bu_4N]PF_6 (0.1 M) was prepared, and an initial cyclic voltammogram (CV) of this solution was collected. A 10 mM stock solution of [^{n}Bu_4N]Cl in acetonitrile was prepared, and 0.8 mL (1 equiv, volume after addition = 5.8 mL) of the stock solution was added to the **1-[V_5FeCl]** sample in a CV cell. The solution was stirred for 1 minute, and a CV was collected. Another 0.8 mL of [^{n}Bu_4N]Cl stock solution was added (2 equiv, volume after addition = 6.6 mL), and a final CV was collected. CV were collected at room temperature with a 200 mV/s scan rate.

Density functional theory calculations:

The calculations were performed using the B3LYP exchange−correlation functional with unrestricted Kohn−Sham wave functions as implemented in the Jaguar electronic structure program.[1] Metal atoms are described using effective core potentials with LACVP-type basis sets, all other atoms are described using the 6-31G++ basis set. Gas phase geometry optimizations and geometry optimizations in solution using the standard Poisson-Boltzmann solvation model were carried out and the resulting minimum energy configurations were used for calculating solvation free energies using the SM 8 solvation model as implemented in the Jaguar code.[2]

Computational redox potentials: One-electron redox process of a redox reaction can be defined in terms of a half-cell reaction as follows:

$$\text{Oxd}^{m} + e- \rightarrow \text{Red}^{m\text{-}1}$$

Where Oxd is an oxidized species and Red is a reduced species. The absolute redox potential $E_{\mathrm{ET}}^{\mathrm{abs}}$ of this redox couple is calculated by eq. (1):

$$E_{\mathrm{ET}}^{\mathrm{abs}} = -\frac{\Delta G^{o}(s)}{F} \quad (1)$$

where $\Delta G^0_{(s)}$ denotes the free energy change in solution and F is Faraday's constant.

To obtain the reduction potentials from calculations, the thermodynamic cycle shown below is used. This is generally defined as a schematic representation of gas-phase and solution phase reactions and the relation between the phases.

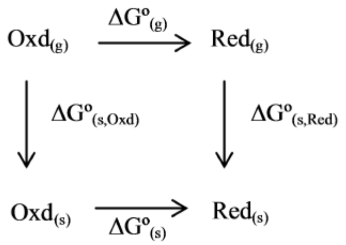

Thermodynamic cycle for the calculation of Gibbs free energies of a one-electron reduction process.

Based on the thermodynamic cycle, $\Delta G^0_{(s)}$ can be expressed as

$$\Delta G^0_{(s)} = \Delta G^0_{(g)} + \Delta G^0_{(s,\ Red)} - \Delta G^0_{(s,\ Oxd)} \qquad (2)$$

Where $\Delta G^0_{(s)}$ is the free energy change in the gas phase, while $\Delta G^0_{(s,Oxd)}$ and $\Delta G^0_{(s,Red)}$ are the solvation free energies of the oxidized and reduced species in acetonitrile, respectively.

Experimentally, the redox potentials are quoted relative to the Fc/Fc$^+$ external standard with a reference potential of $E^{\text{abs}}_{\text{exp,RE}}$ = 0.64 V relative to the standard hydrogen electrode.[2] The theoretical redox potentials with respect to the experimental reference are calculated using equation (3):

$$E^{\text{calc}}_{\text{ET}}(\text{V } vs. \text{ Fc/Fc}^+) = E^{\text{abs}}_{\text{ET}} - E^{\text{abs}}_{\text{SHE}} - E^{\text{abs}}_{\text{exp,RE}} \quad (3)$$

Where $E^{\text{abs}}_{\text{SHE}}$ denotes the absolute electrode potential of the standard hydrogen electrode.

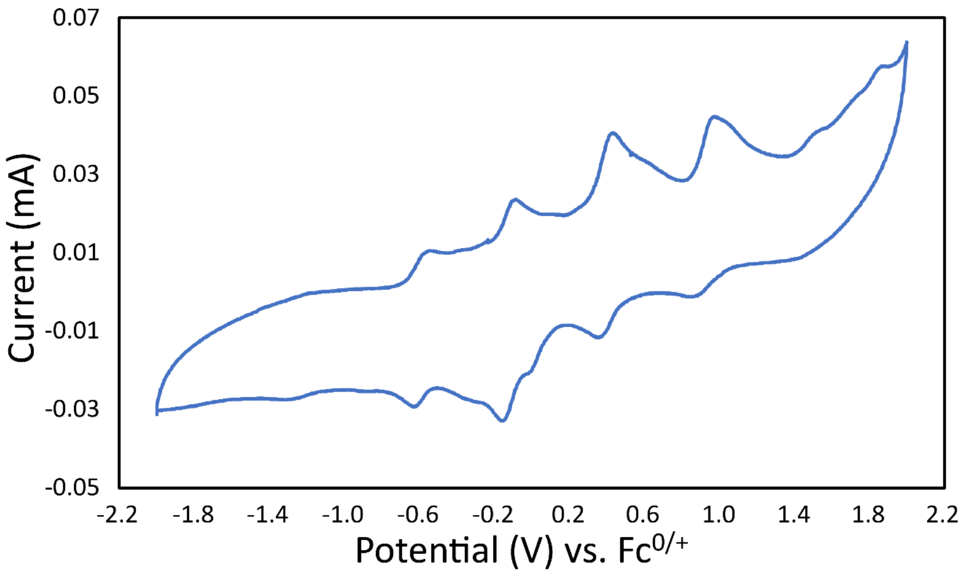

Figure S1. Cyclic voltammogram of **1-[V_5FeCl]** collected in acetonitrile scanning from -2 to 2 V (scan rate = 200 mV/s, 0.1 M (^{n}Bu$_4$N)PF$_6$ as supporting electrolyte, referenced to Fc$^{0/+}$ redox couple). Impurities from oxidative decomposition are formed electrochemically as the window is expanded.

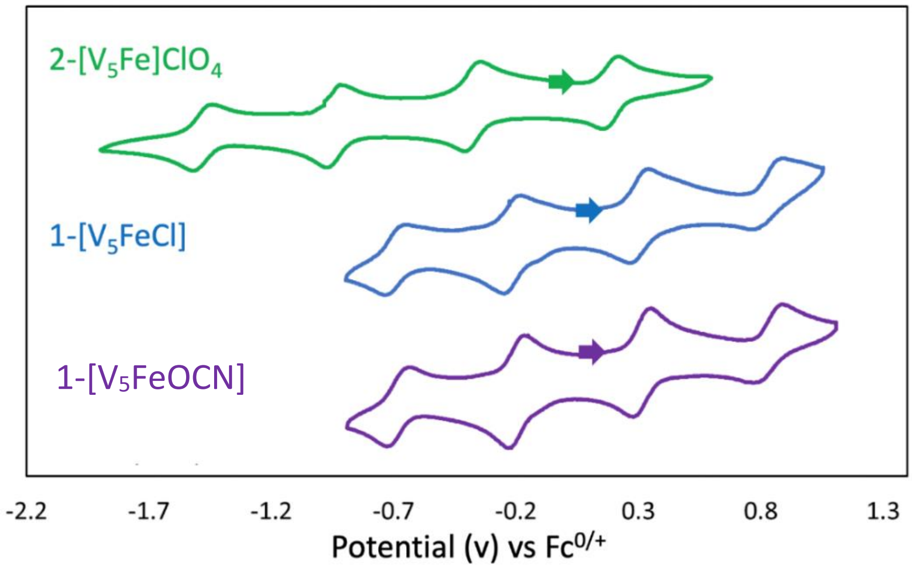

Figure S2. Cyclic voltammograms of **2-[V_5Fe]ClO_4** (top, green), **1-[V_5FeCl]** (middle, blue), and **1-[V_5FeOCN]** (bottom, purple) collected in acetonitrile (0.1 M [^{n}Bu$_4$N]PF_6 as supporting electrolyte, referenced to $Fc^{0/+}$ redox couple).

Table S1. The half wave potentials ($E_{1/2}$) values for **1-[V_5FeCl]**, **2-[V_5Fe]ClO_4,** and **1-[V_5FeOCN]** from the cyclic voltammograms in Figure SX (acetonitrile, 0.1 M (^{n}Bu$_4$N)PF_6 as supporting electrolyte, referenced to $Fc^{0/+}$ redox couple).

			**Half Wave Potential ($E_{1/2}$, V) vs. $Fc^{0/+}$ in acetonitrile**			
**Cluster**	**CN***	**Event 0** $V^{III}V^{IV}_4Fe^{III}$/ $V^{IV}_5Fe^{III}$	**Event 1** V^{IV}_5 Fe^{III} / $V^{IV}_4V^V$ Fe^{III}	**Event 2** $V^{IV}_4V^V$ Fe^{III} / $V^{IV}_3V^V_2$ Fe^{III}	**Event 3** $V^{IV}_3V^V_2$ Fe^{III} / $V^{IV}_2V^V_3$ Fe^{III}	**Event 4** $V^{IV}_2V^V_3$ Fe^{III} / $V^{IV}V^V_4$ Fe^{III}
**1-[V_5FeOCN]**	6	---	-0.67	+0.19	+0.32	+0.84
**1-[V_5FeCl]**	6	---	-0.68	-0.21	+0.31	+0.83
**2-[V_5Fe]ClO_4**	5	-1.46	-0.94	-0.36	+0.21	---

*Coordination number around the heteroion

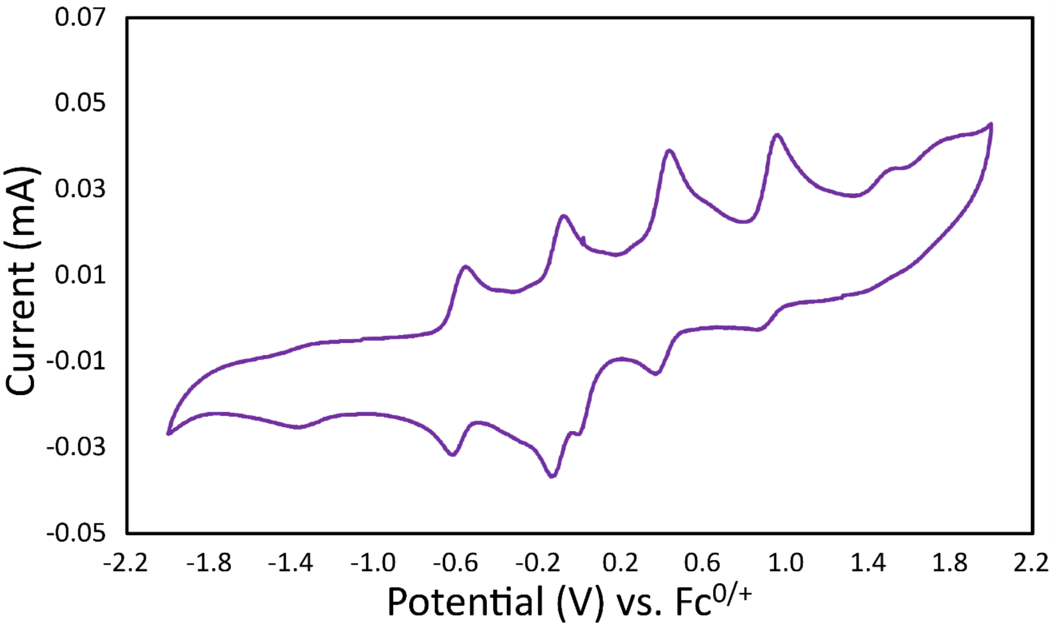

Figure S3. Cyclic voltammogram of **1-[V_5FeOCN]** collected in acetonitrile (scan rate = 200 mV/s, 0.1 M (^{n}Bu$_4$N)PF_6 as supporting electrolyte, referenced to $Fc^{0/+}$ redox couple). Like **1-[V_5FeCl]** (Figure S1), impurities are formed electrochemically as the window of the experiment is expanded.

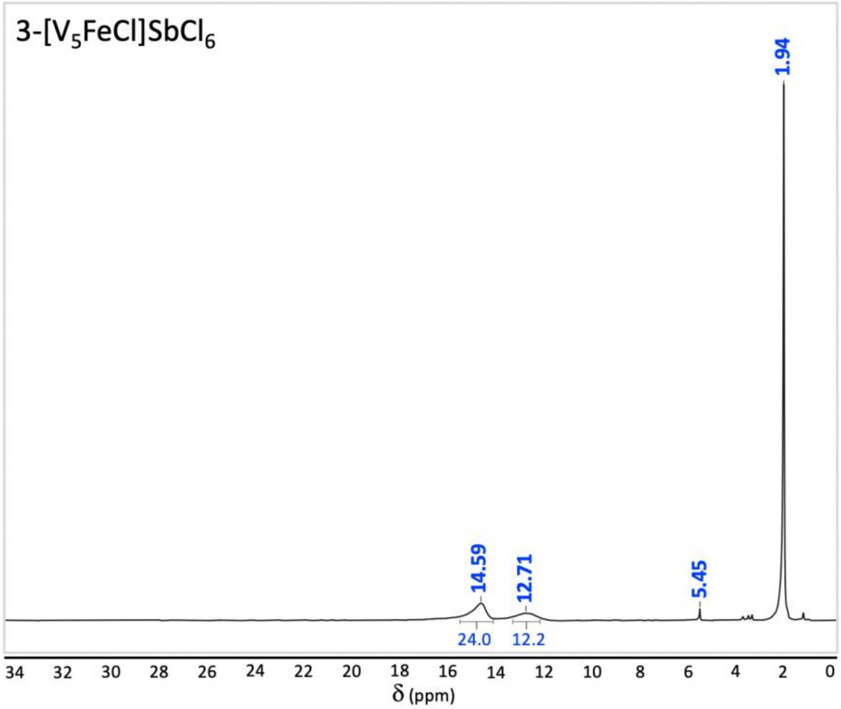

Figure S4. ^{1}H NMR spectrum of **3-[V_5FeCl]SbCl$_6$** (CD_3CN, 21 °C, 400 MHz). Peaks: 14.59 (24 H, -OCH_3), 12.71 (12 H, -OCH_3), 5.45 (DCM), 1.94 (CH_3CN, reference) ppm.

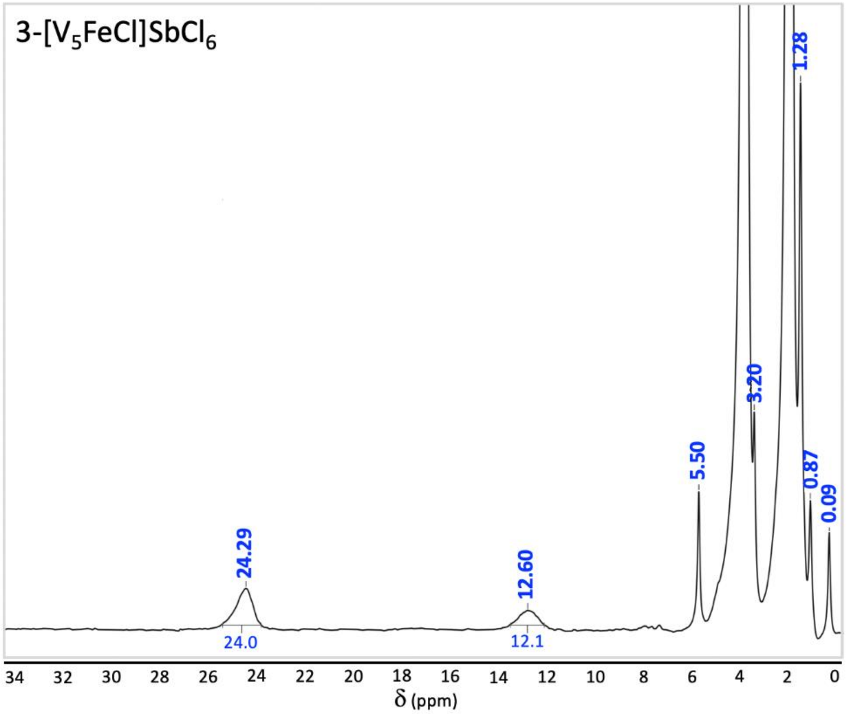

Figure S5. ^{1}H NMR spectrum of **3-[V_5FeCl]SbCl_6** (THF-d_8, 21 °C, 400 MHz). This spectrum matched that of previously reported [$V_5O_6(OMe)_{12}$FeCl]SO_3CF_3.[3] Peaks: 24.29 (24 H, -OCH_3), 12.60 (12 H, -OCH_3), 5.50 (DCM), 3.58 (THF, reference), 3.20, 1.72 (THF), 1.28 (pentane), 0.87 (pentane), 0.09 (grease) ppm.

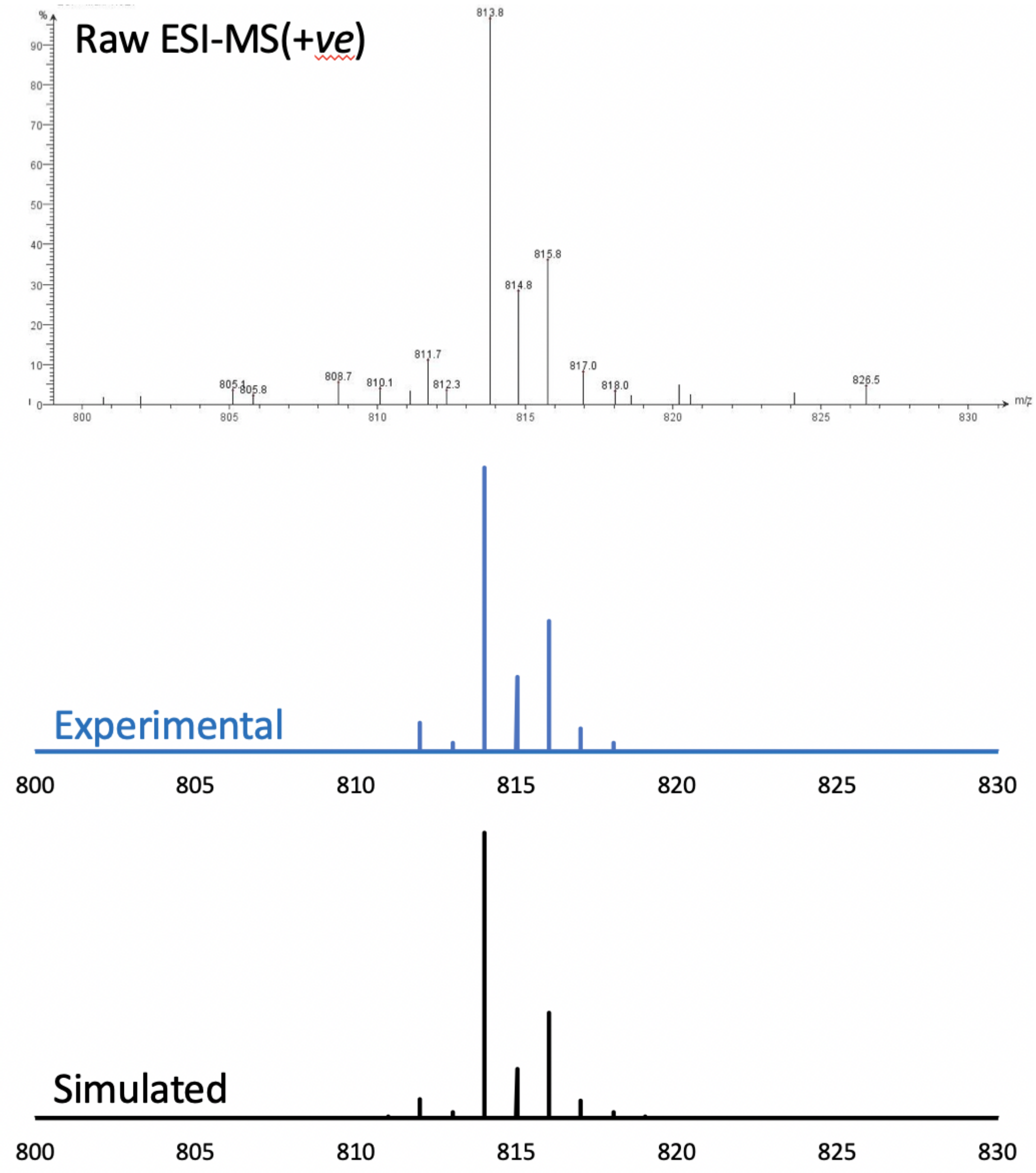

Figure S6. ESI-MS (+*ve*, acetonitrile) of **3-[V_5FeCl]SbCl_6**.

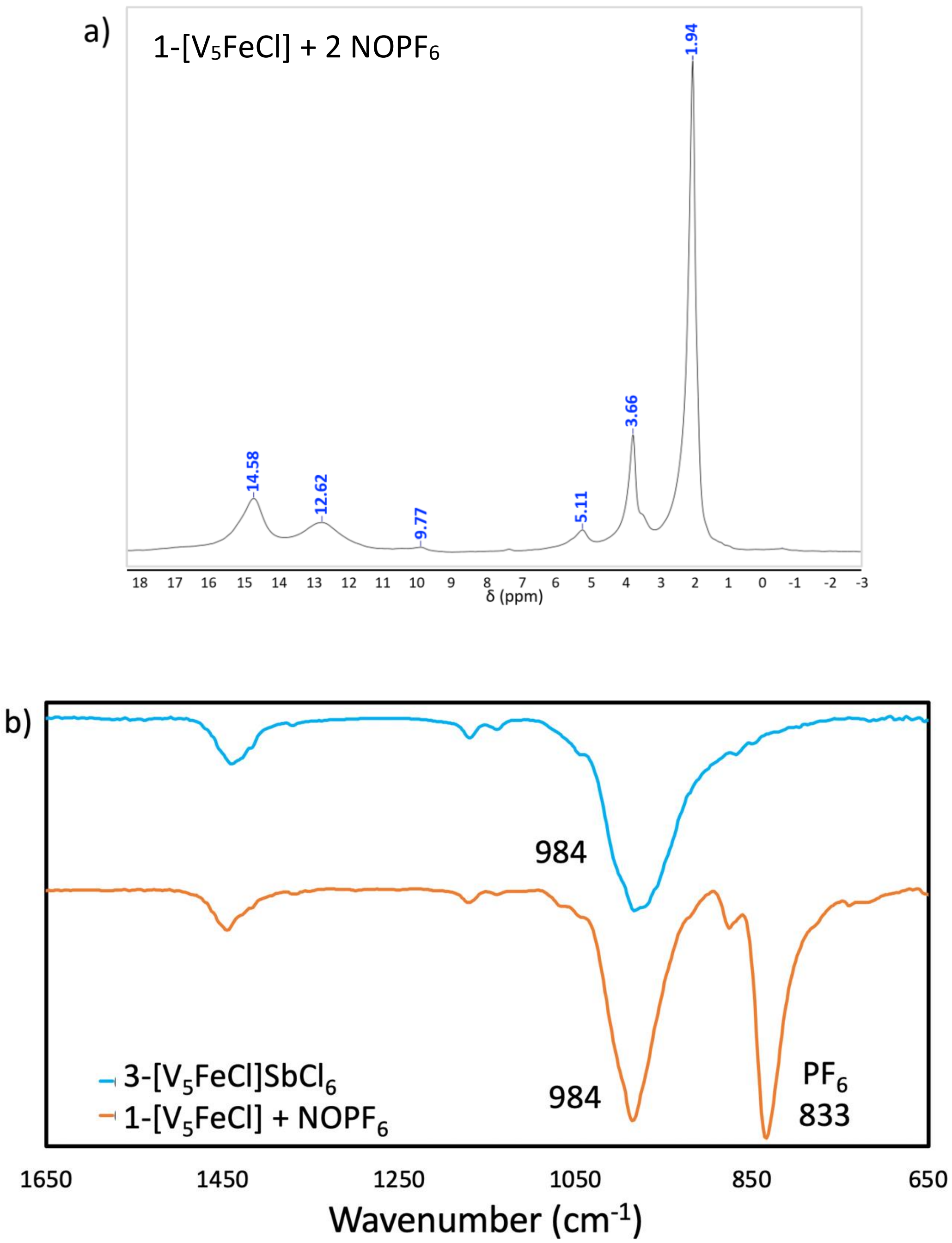

Figure S7. a) ^{1}H NMR spectrum (CD_3CN, 21 °C, 400 MHz) ; peaks: 14.58 (-OCH_3), 12.62 (-OCH_3), 9.77, 5.11 (DCM), 3.66, 1.94 (CH_3CN, reference) ppm.and b) infrared spectrum of the crude reaction mixture of **1-[V_5FeCl]** + 2 equiv. $NOPF_6$ in dichloromethane.

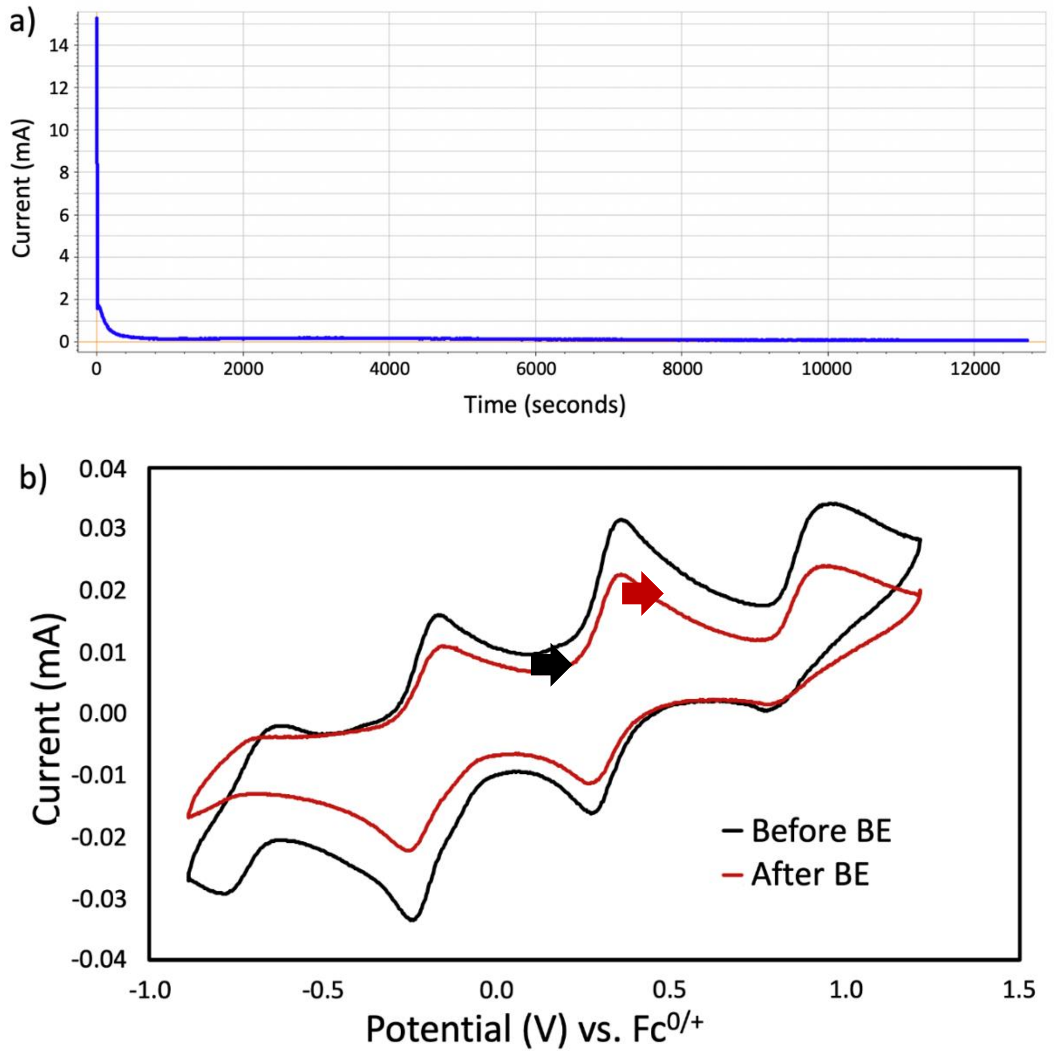

Figure S8. a) Current versus time trace and b) cyclic voltammograms (scan rate = 200 mV/s) before and after bulk oxidation of **1-[V_5FeCl]** to +1.3 V (acetonitrile, 0.1 M [^{n}Bu$_4$N]PF$_6$ as supporting electrolyte, referenced to Fc$^{0/+}$ redox couple). Arrow indicate direction of scan and the location of the open circuit potential. The decrease in the current observed after bulk oxidation is indicative of decomposition.

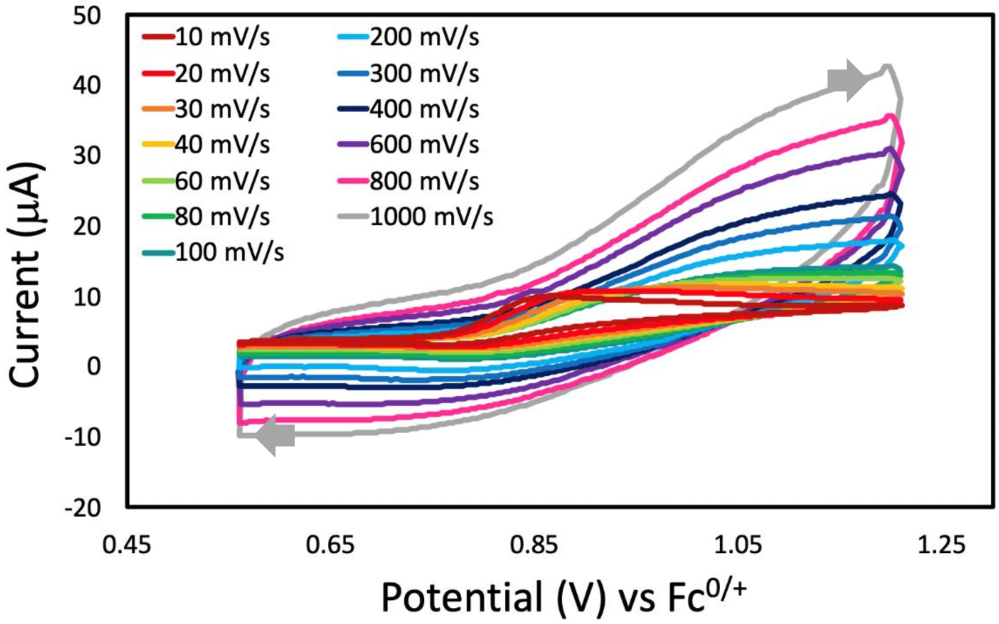

Figure S9. Cyclic voltammograms (CV) of the most oxidizing event of **1-[V_5FeCl]** (acetonitrile, 0.1 M (^{n}Bu$_4$N)PF$_6$ as supporting electrolyte, referenced to Fc$^{0/+}$ redox couple) at varying scan rates.

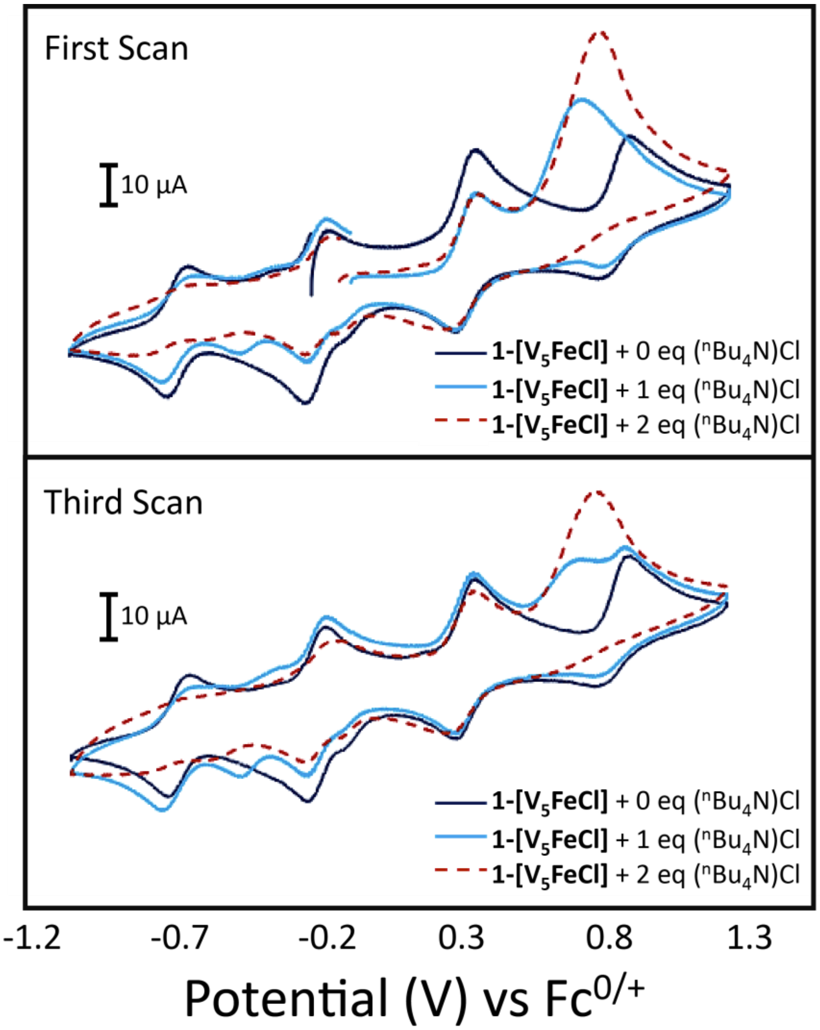

Figure S10. Scan 1 (top) and scan 3 (bottom) of the cyclic voltammograms (CV) following titration of 0 equiv (dark blue), 1 equiv (light blue), and 2 equiv (red, dotted) of an acetonitrile solution of [^{n}Bu$_4$N]Cl into **1-[V$_5$FeCl]** (acetonitrile, 200 mV/s, 0.1 M (^{n}Bu$_4$N)PF$_6$ as supporting electrolyte, referenced to Fc$^{0/+}$ redox couple). Heterogeneous chloride oxidation is observed at 0.79 V when [^{n}Bu$_4$N]Cl is present. Redox features associated with **1-[V$_5$FeCl]** are retained upon addition of [^{n}Bu$_4$N]Cl.

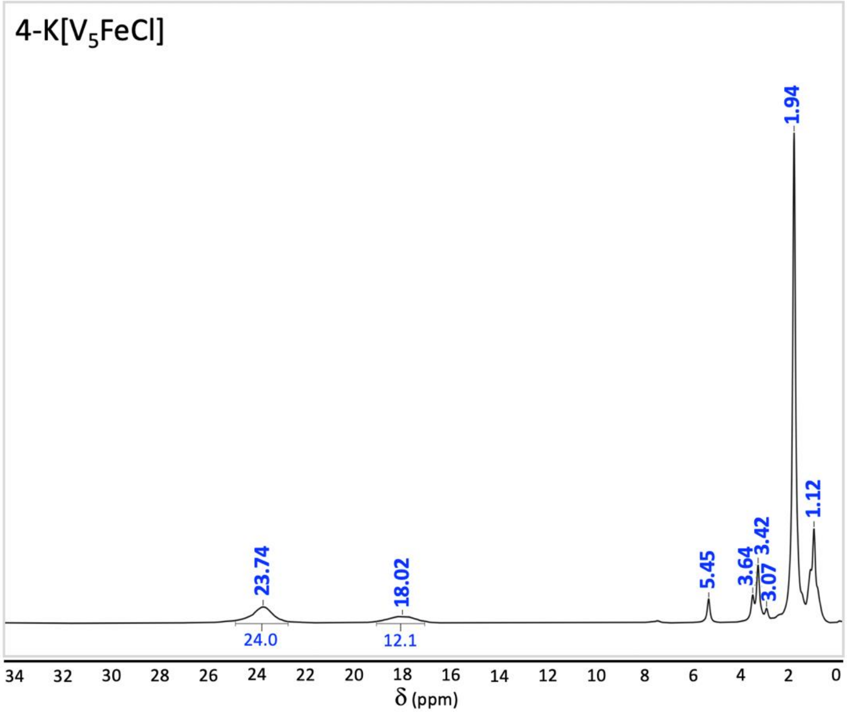

Figure S11. ^{1}H NMR spectrum of **4-K[V_5FeCl]** (CD_3CN, 21 °C, 400 MHz). Peaks: 23.74 (24 H, -OCH_3), 18.02 (12 H, -OCH_3), 5.45 (DCM), 3.64 (THF), 3.42 (Et_2O), 1.94 (CH_3CN, reference), 1.12 (Et_2O) ppm.

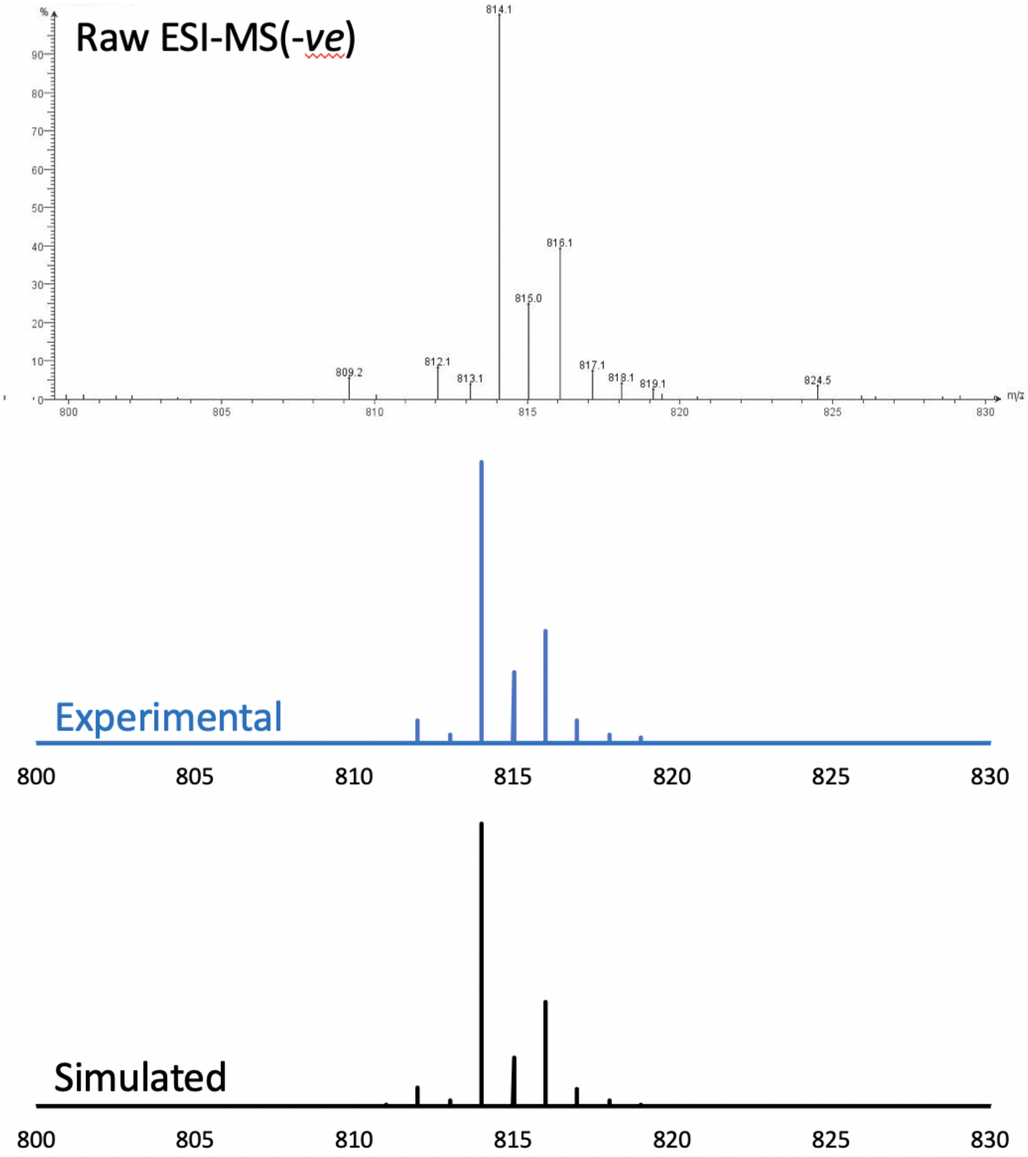

Figure S12. ESI-MS (-*ve*, acetonitrile) of **4-K[V_5FeCl]**.

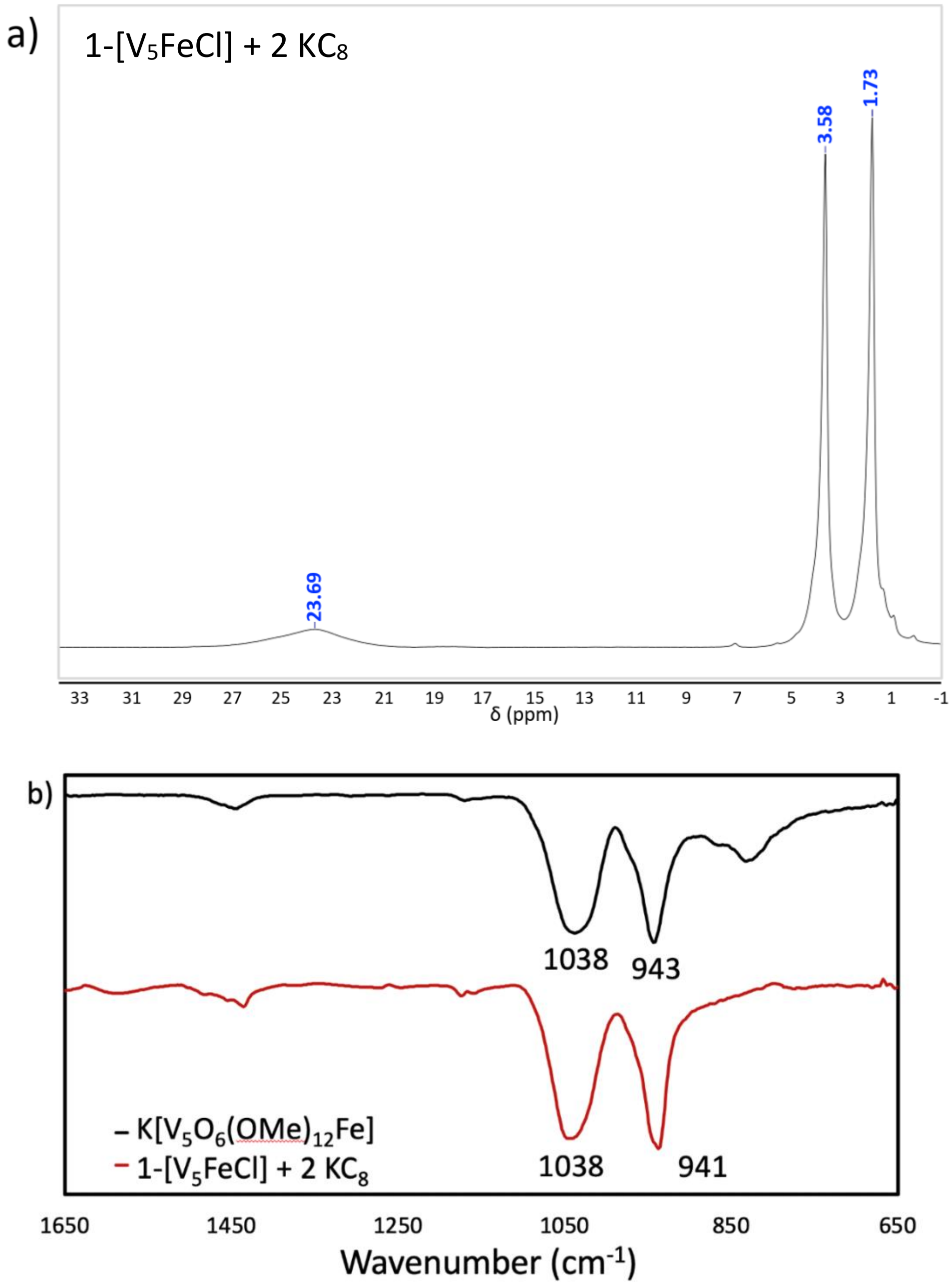

Figure S13. a) ^{1}H NMR spectrum (THF-d_8, 21 °C, 400 MHz) ; peaks: 23.69 (-OCH_3), 3.58 (THF, reference), 1.73 (THF) ppm.and b) infrared spectrum of the crude reaction mixture of **1-[V_5FeCl]** + 2 equiv. KC_8 in tetrahydrofuran. The spectra match that of previously reported $K_2[V_5O_6(OMe)_{12}Fe]$.[1]

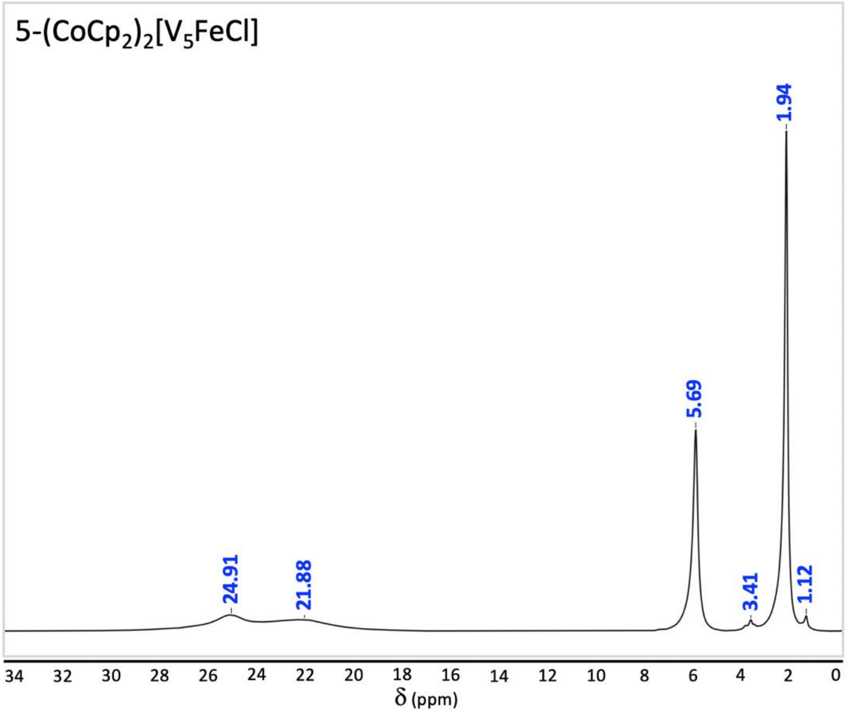

Figure S14. ^{1}H NMR spectrum of **5-(CoCp$_2$)$_2$[V$_5$FeCl]** (CD_3CN, 21 °C, 400 MHz). Peaks: 24.91 (-OCH^3), 21.88 (-OCH_3), 5.69 ($CoCp_2^+$), 3.41 (Et_2O), 1.94 (CH_3CN, reference), 1.12 (Et_2O) ppm. Bridging methoxide peaks could not be reliably integrated due to overlap.

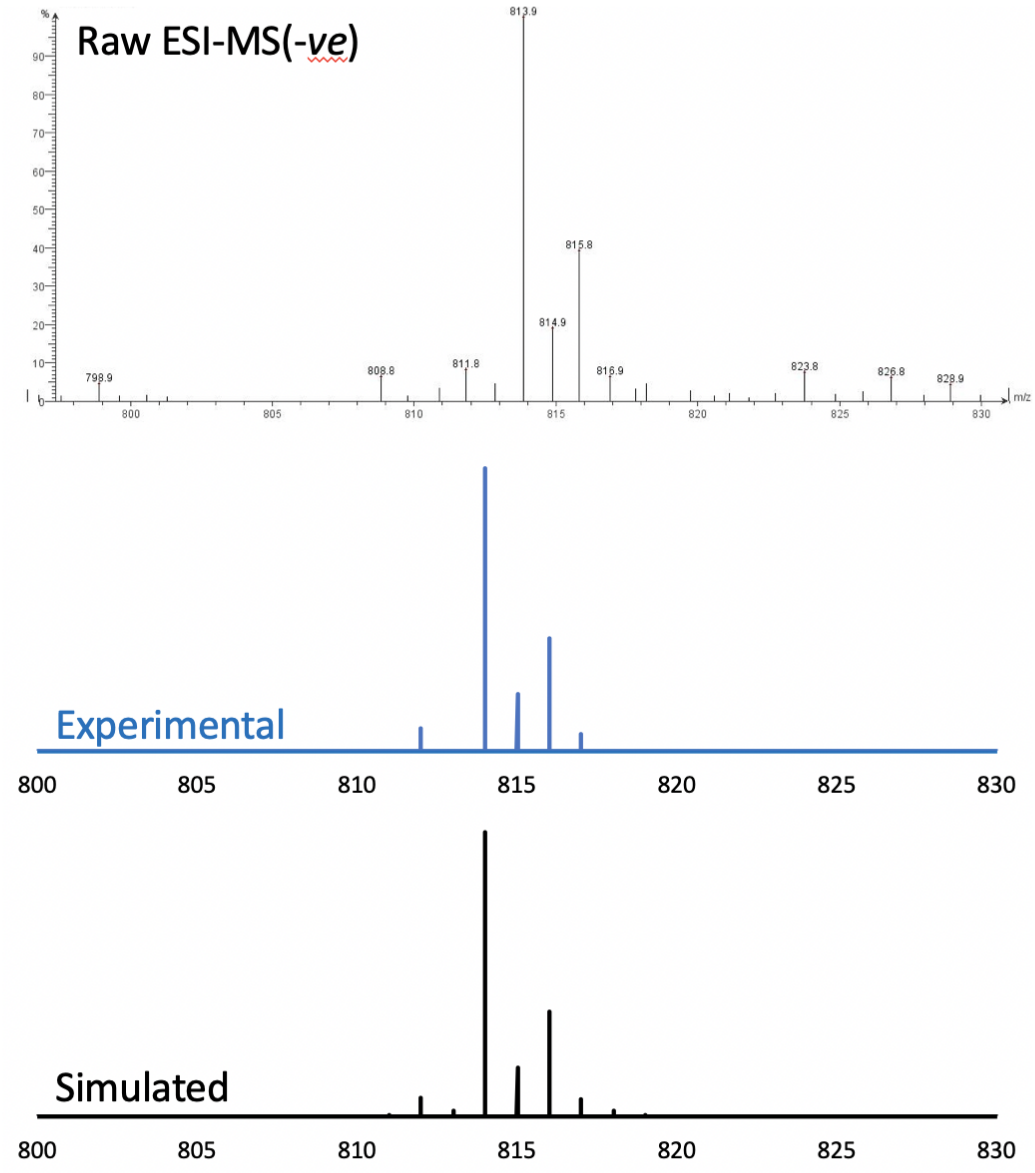

Figure S15. ESI-MS (-*ve*, acetonitrile) of **5-$(CoCp_2)_2[V_5FeCl]$**.

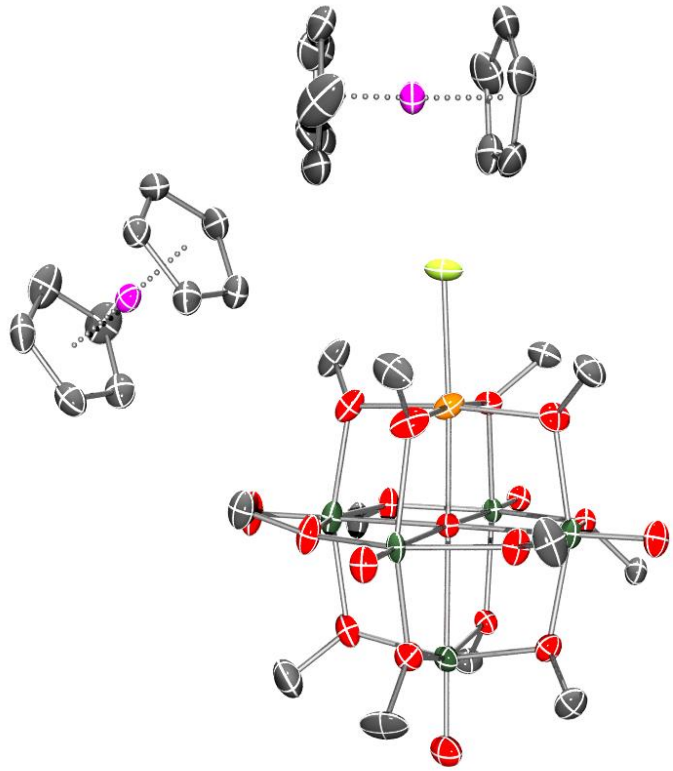

Figure S16. Molecular structure of **5-$(CoCp)_2$[V_5FeCl]** shown with 40% probability ellipsoids. The atoms are color-coded as follows: iron, orange; vanadium, green; cobalt, pink; oxygen, red; chloride, yellow; carbon, gray. Hydrogen atoms and co-crystallized dichloromethane were removed for clarity.

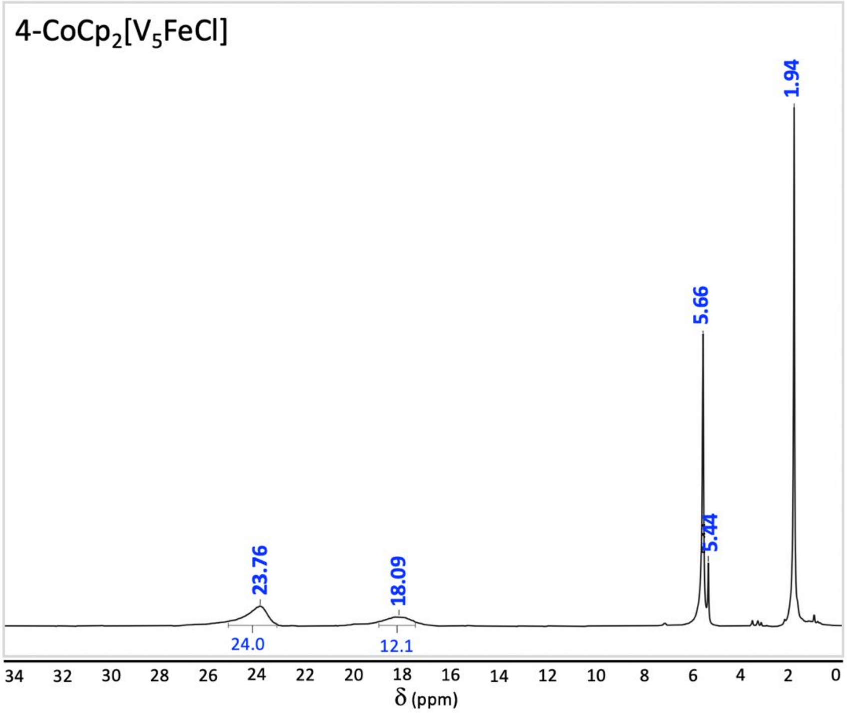

Figure S17. ^{1}H NMR spectrum of **4-CoCp$_2$[V$_5$FeCl]** (CD_3CN, 21 °C, 400 MHz). Peaks: 23.76 (24 H, -OCH_3), 18.09 (12 H, -OCH_3), 5.66 ($CoCp_2^+$), 5.44 (DCM), 1.94 (CH_3CN, reference) ppm.

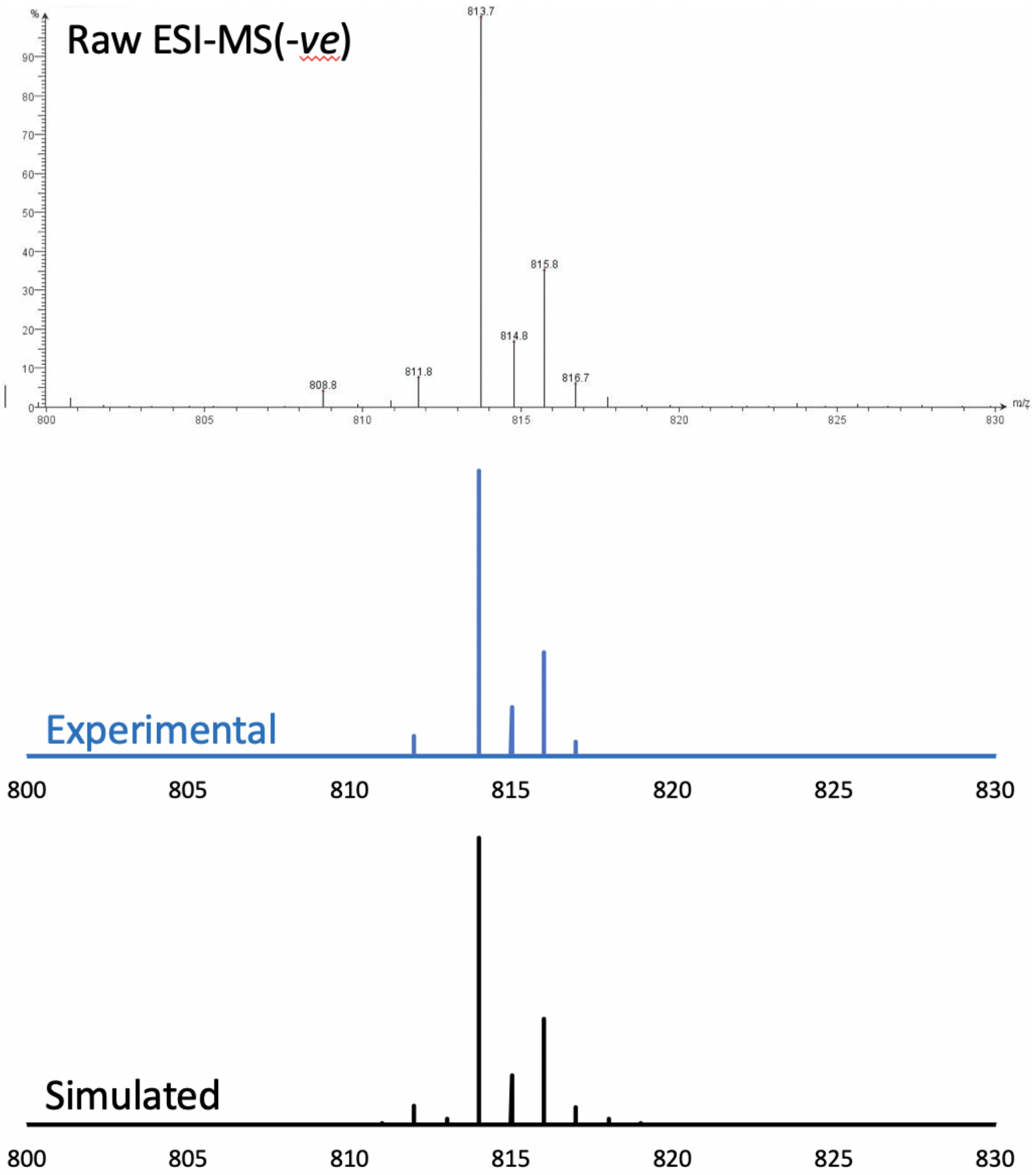

Figure S18. ESI-MS (-*ve*, acetonitrile) of **4-CoCp$_2$[V$_5$FeCl]**.

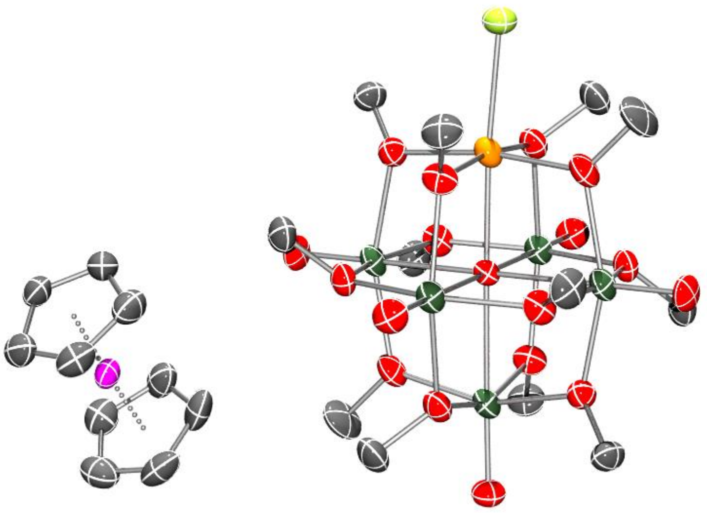

Figure S19. Molecular structure of **4-CoCp$_2$[V$_5$FeCl]** shown with 40% probability ellipsoids. The atoms are color-coded as follows: iron, orange; vanadium, green; cobalt, pink; oxygen, red; chloride, yellow; carbon, gray. Hydrogen atoms and co-crystallized tetrahydrofuran were removed for clarity.

Table S2. Crystallographic parameters for**4-CoCp$_2$[V$_5$FeCl]** and **5-(CoCp$_2$)$_2$[V$_5$FeCl]**.

Empirical formula	$C_{26}H_{54}ClCoFeO_{19}V_5$	$C_{34}H_{60}Cl_5Co_2FeO_{18}V_5$
Formula weight	1075.62	1362.48
Temperature	100.00(10) K	100.00(10)K
Wavelength	1.54184 Å	1.54184 Å
Crystal system	Triclinic	Monoclinic
Space group	*P*-1	$P2_1/n$
Unit cell dimensions	a = 10.0421(3) Å<br>b = 10.4129(4) Å<br>c = 21.7410(4) Å<br>α = 92.613(2)°<br>β = 96.439(2)°<br>γ = 117.412(4)°	a = 15.3662(2) Å<br>b = 14.6105(2) Å<br>c = 22.0713(3) Å<br>α = 90°<br>β = 92.7580(10)°<br>γ = 90°
Volume	1993.19(12) Å^3	4949.44(12) Å^3
Z	2	4
Reflections collected	29699	49417
Independent reflections	8308	10429
Goodness-of-fit on F2	1.052	1.076
Final R indices [I>2sigma(I)]	R1 = 0.0720, wR2 = 0.1925	R1 = 0.0453, wR2 = 0.1209

Table S3. Values for the *v*(O_b-CH_3) and *v*(V=O_t) bands in the FT-IR spectra of **1-[V_5FeCl]**, and **3-[V_5FeCl]$SbCl_6$, 4-K[V_5FeCl]**, and **5-($CoCp_2$)$_2$[V_5FeCl]**.

Cluster	Oxidation State Distribution	*v*(O_b-OCH_3) (cm^{-1})	*v*(V=O_t) (cm^{-1})
**5-[$CoCp_2$]$_2$[V_5FeCl]**	$V^{IV}{}_5Fe^{III}$	1043	941
**4-K[V_5FeCl]**	$V^{IV}{}_4V^{V}Fe^{III}$	1018	957
**1-[V_5FeCl]**	$V^{IV}{}_3V^{V}{}_2Fe^{III}$	1003	969
**3-[V_5FeCl][$SbCl_6$]**	$V^{IV}{}_2V^{V}{}_3Fe^{III}$	---	984

Table S4. Absorbance features observed in the electronic absorption spectra of **5-[$CoCp_2$]$_2$[V_5FeCl]**, **4-[$CoCp_2$][V_5FeCl]**, **1-V_5FeCl**, and **3-[V_5FeCl][$SbCl_6$]** and the assigned electronic transition.

		Wavelength (Molar Absorptivity), nm (1 x 10^3 M^{-1} cm^{-1})			
Cluster	Oxidation State Distribution	Cl: → Fe^{III}	$d_{xy}(V^{IV}) \rightarrow d_{x^2-y^2}(V^{V})$	$d_{xy}(V^{IV}) \rightarrow d_{xy}(V^{V})$	$d_{xy}(V^{IV}) \rightarrow d_{x^2-y^2}(V^{IV})$
**5-[$CoCp_2$]$_2$[V_5FeCl]**	$V^{IV}{}_5Fe^{III}$	296 (7.16)	---	---	598 (0.24)
**4-K[V_5FeCl]**	$V^{IV}{}_4V^{V}Fe^{III}$	300 (9.71)	382 (3.65)	992 (0.55)	---
**1-[V_5FeCl]**	$V^{IV}{}_3V^{V}{}_2Fe^{III}$	308 (11.1)	382 (8.16)	990 (0.95)	---
**3-[V_5FeCl][$SbCl_6$]**	$V^{IV}{}_2V^{V}{}_3Fe^{III}$	316 (9.92)	382 (8.01)	992 (0.66)	---

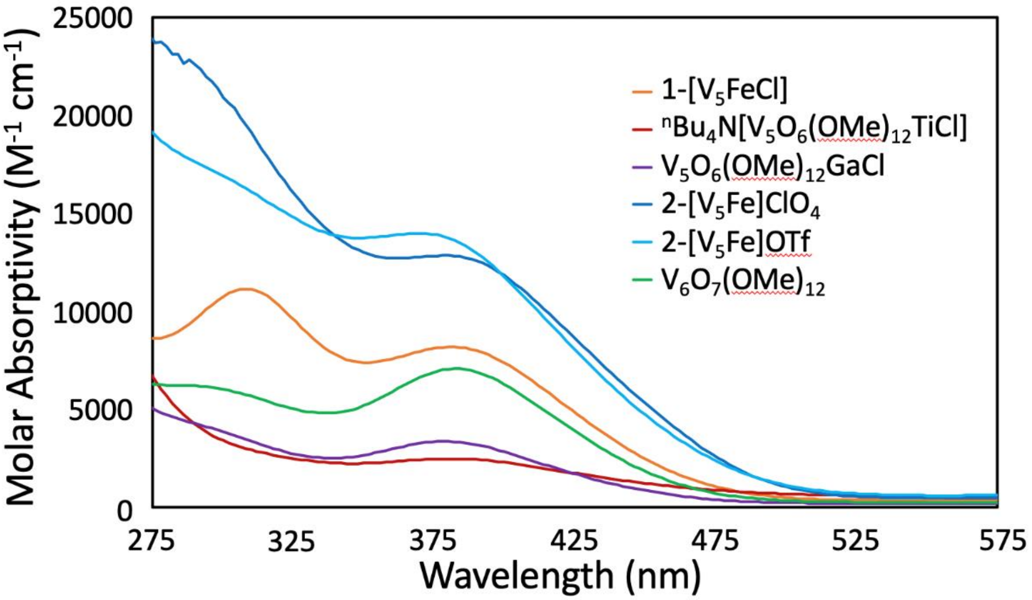

Figure S20. Electronic absorption spectra (collected in acetonitrile) of **1-[V_5FeCl]**, $^nBu_4N[V_5O_6(OMe)_{12}TiCl]$, $V_5O_6(OMe)_{12}GaCl$, **2-[V_5Fe]ClO_4**, **2-[V_5Fe]OTf**, and $V_6O_7(OMe)_{12}$. The Cl: → M LMCT (M = Fe^{III}) is located at 308 nm for **1-[V_5FeCl]**. Notably, this feature is absent in $V_5O_6(OMe)_{12}GaCl$ and the other POV-alkoxide clusters that do not contain chloride ions.

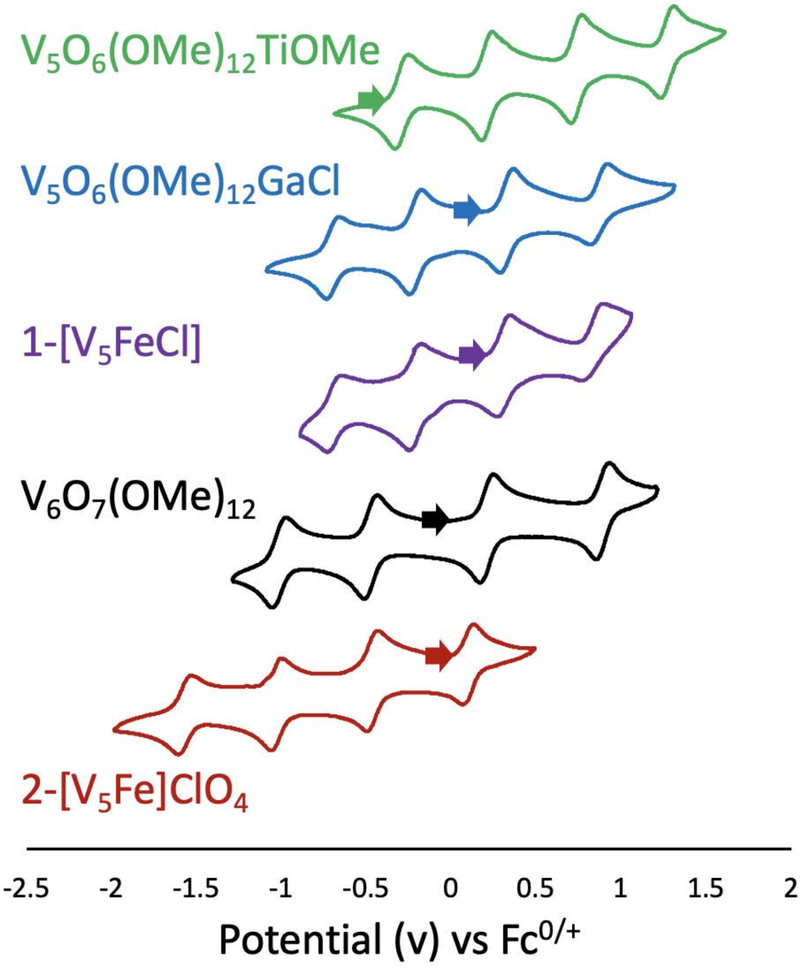

Figure S21. Cyclic voltammograms of $[V_5O_6(OMe)_{12}TiOMe]^{-1}$ (Green), $[V_5O_6(OMe)_{12}GaCl]$ (Blue), **1-[V$_5$FeCl]** (Purple), $V_6O_7(OMe)_{12}$ (Black), and **2-[V$_5$Fe]ClO$_4$** (Red) collected in acetonitrile (0.1 M ($^nBu_4N)PF_6$ as supporting electrolyte, referenced to $Fc^{0/+}$ redox couple). The $V_5O_6(OMe)_{12}TiOMe$ cluster is representative of the group(IV)-functionalized clusters, since the half-wave potentials for these clusters are similar.

Table S5. Aqueous pKa values[4] for the installed heteroion (M) and the half wave potentials ($E_{1/2}$) values for the POV-alkoxide clusters (acetonitrile, 0.1 M ($^nBu_4N)PF_6$ as supporting electrolyte, referenced to $Fc^{0/+}$ redox couple).

				**Half Wave Potential ($E_{1/2}$, V) vs. $Fc^{0/+}$ in acetonitrile**			
**Cluster**	**Heteroion (M)**	**CN***	**pKa $M(aquo)^{n+}$**	**Event 1** $V^{IV}_5M/V^{IV}_4V^VM$	**Event 2** $V^{IV}_4V^VM/V^{IV}_3V^V_2M$	**Event 3** $V^{IV}_3V^V_2M/V^{IV}_2V^V_3M$	**Event 4** $V^{IV}_2V^V_3M/V^{IV}V^V_4M$
$V_5O_6(OMe)_{12}TiOMe$	Ti^{4+}	6	1.8	-0.38	+0.20	+0.73	+1.29
$V_5O_6(OMe)_{12}ZrOMe$	Zr^{4+}	6	1.2	-0.32	+0.16	+0.70	+1.29
$V_5O_6(OMe)_{12}HfOMe$	Hf^{4+}	6	1.1	-0.34	+0.17	+0.71	+1.25
**1-[V$_5$FeCl]**	Fe^{3+}	6	2.7	-0.68	-0.21	+0.31	+0.83
$V_5O_6(OMe)_{12}GaCl$	Ga^{3+}	6	3.0	-0.66	-0.20	+0.34	+0.88
$V_6O_7(OMe)_{12}$	V^{4+}	6	4.8	-0.81	-0.31	+0.21	+0.76
**2-[V$_5$Fe]ClO$_4$**	Fe^{3+}	5	2.7	-0.94	-0.36	+0.21	---

*Coordination number around the heteroion

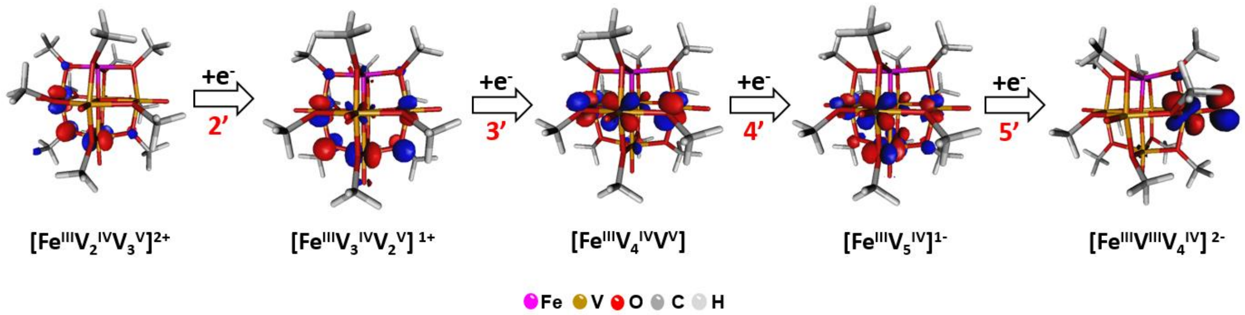

Figure S22: Frontier molecular orbital plots showing the highest occupied molecular orbitals (HOMO) for the one-electron oxidized species **2-[V$_5$Fe]ClO$_4$** (left) as well as the one-, two-, three-, and four-electron reduced species. Red numbers mark the reduction steps experimentally observed in cyclic voltammetry (Table 1).

Table S6. Atomic spin densities from Mulliken analysis for the complete set of redox-isomers of **1-[V$_5$FeCl]**.

Cluster	Atomic spin densities					
	Fe	V1	V2	V3	V4	V5
**[Fe^{III}V^{IV}V$_4$^{V}Cl]$^{2+}$**	4.03	-0.01	-0.01	-0.01	1.12	0.02
**[Fe^{III}V$_2$^{IV}V$_3$^{V}Cl]$^{1+}$**	4.06	0.03	0.00	0.01	1.11	1.11
**[Fe^{III}V$_3$^{IV}V$_2$^{V}Cl]**	4.07	1.11	-0.02	-1.10	-0.02	-1.09
**[Fe^{III}V$_4$^{IV}V^{V}Cl]$^{1-}$**	4.09	0.02	-1.11	-1.11	1.10	1.10
**[Fe^{III}V$_5$^{IV}Cl]$^{2-}$**	4.11	1.11	1.11	1.11	1.11	1.11

Table S7. Atomic spin densities from Mulliken analysis for the complete set of redox-isomers of **2-[V$_5$Fe]ClO$_4$**.

Cluster	Atomic spin densities					
	Fe	V1	V2	V3	V4	V5
**[Fe^{III}V$_2$^{IV}V$_3$^{V}]$^{2+}$**	4.05	1.11	0.00	0.01	0.01	1.11
**[Fe^{III}V$_3$^{IV}V$_2$^{V}] $^{1+}$**	4.04	0.04	1.11	0.03	1.11	1.10
**[Fe^{III}V$_4$^{IV}V^{V}]**	4.04	1.10	1.10	1.10	1.10	0.11
**[Fe^{III}V$_5$^{IV}]$^{1-}$**	4.03	1.10	1.11	1.11	1.11	1.11
**[Fe^{III}V^{III}V$_4$^{IV}] $^{2-}$**	4.02	1.94	1.11	1.11	1.11	1.13

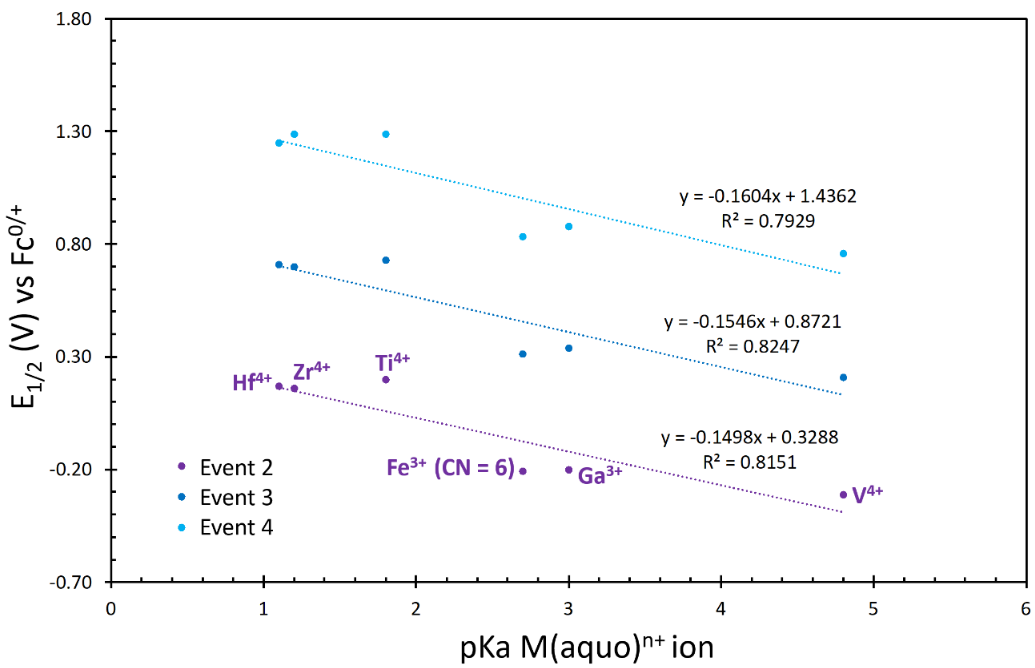

Figure S23. Plot of the half wave potential of $V^{IV}{}_4V^{V}M/V^{IV}{}_3V^{V}{}_2M$ (Event 2), $V^{IV}{}_3V^{V}{}_2M/V^{IV}{}_2V^{V}{}_3M$ (Event 3), and $V^{IV}{}_2V^{V}{}_3M/V^{IV}V^{V}{}_4M$ (Event 4) redox couples (M = Hf^{4+}, Zr^{4+}, Ti^{4+}, Fe^{3+}, Ga^{3+}, and V^{4+})[5] of POV alkoxide clusters (acetonitrile, 0.1 M ($^nBu_4N)PF_6$ as supporting electrolyte, referenced to $Fc^{0/+}$) versus the pKa of the aqueous heteroion (M). CN refers to the coordination number around Fe^{3+}. Complex **2-[V$_5$Fe]ClO$_4$** (CN = 5) is excluded from this analysis.

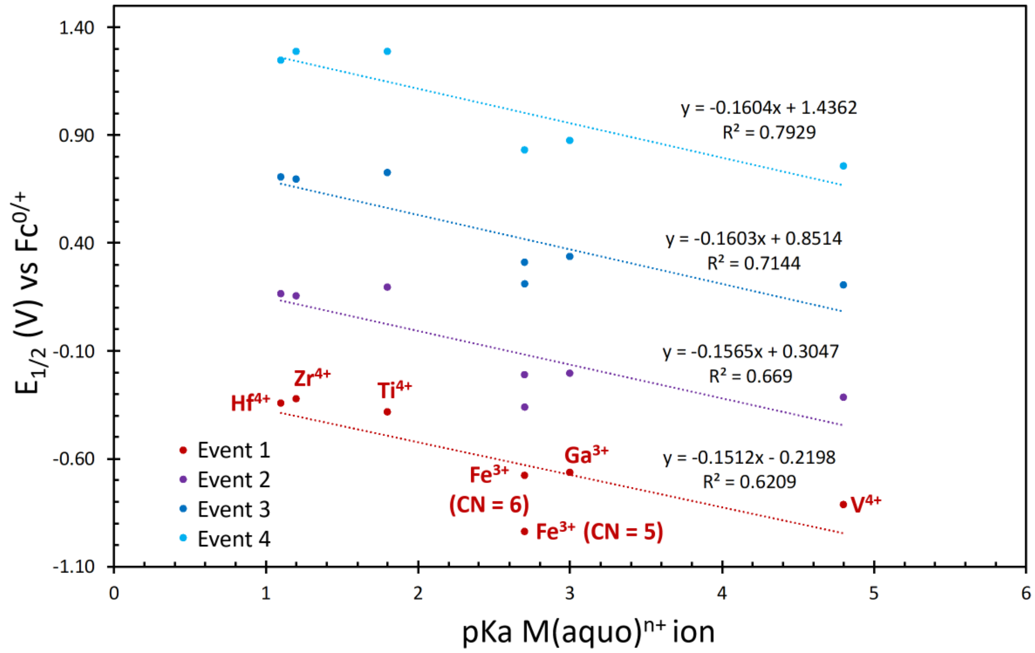

Figure S24. Plot of the half wave potential of $V^{IV}{}_5M/V^{IV}{}_4V^{V}M$ (Event 1), $V^{IV}{}_4V^{V}M/V^{IV}{}_3V^{V}{}_2M$ (Event 2), $V^{IV}{}_3V^{V}{}_2M/V^{IV}{}_2V^{V}{}_3M$ (Event 3), and $V^{IV}{}_2V^{V}{}_3M/V^{IV}V^{V}{}_4M$ (Event 4) redox couples (M = Hf^{4+}, Zr^{4+}, Ti^{4+}, Fe^{3+}, Ga^{3+}, and V^{4+})[5] of POV alkoxide clusters (acetonitrile, 0.1 M ($^nBu_4N)PF_6$ as supporting electrolyte,

referenced to $Fc^{0/+}$) versus the pKa of the aqueous heteroion (M). CN refers to the coordination number around Fe^{3+}. Complex **2-[V$_5$Fe]ClO$_4$** (CN = 5) is included from this analysis.

Table S8. Geometry Optimized Coordinates of **[$V^{IV}V^{V}{}_4Fe^{III}Cl]^{2+}$**

Fe1	7.4209747263	7.5103184007	13.9782820882
V2	9.9081036639	8.2355443227	12.1376487997
V3	7.2358415716	10.0636448660	12.0446544930
V4	5.4936536440	7.4038586972	11.4526939466
V5	8.1528615523	5.5920044619	11.5200816960
V6	7.9837080868	8.1454180106	9.5439252658
Cl7	7.0820736741	7.3325917938	16.2001240407
O8	7.6873916277	7.8483732714	11.7967753376
O9	9.4006866751	8.0324927265	13.9338095587
O10	7.0169620608	9.5956273457	13.8081610958
O11	5.4377194545	7.3562260915	13.2856264931
O12	7.7654031548	5.6584400376	13.4652826766
O13	9.9904355185	6.3711019919	11.9030980189
O14	9.1601880026	10.0806868822	12.2432184861
O15	5.4998519838	9.3975071290	11.5378901587
O16	6.1282670438	5.6763767499	11.1966435981
O17	9.8097495594	8.4724465943	10.2058609758
O18	7.6421760774	9.9730375562	10.0998808827
O19	6.1291745358	7.8067841293	9.5622040538
O20	8.3664193515	6.3127393729	9.6140717076
O21	11.4291281250	8.5696657099	12.3571267490
O22	6.9494018122	11.6093819690	12.1213482900
O23	3.9779173999	7.2136895260	11.0761403792
O24	8.4701633133	4.0626998160	11.2641639881
O25	8.2521821256	8.3999614178	8.0168537222
C26	10.2439730718	8.1158362715	15.1422030663
C27	6.5825892112	10.4463302775	14.9346626600
C28	4.2292900441	7.4915463870	14.1263645682
C29	7.3294841089	4.4720648364	14.2260023220
C30	11.0688805739	5.5027506718	12.4247564880
C31	9.8845394597	11.1692782155	12.9280241581
C32	4.5624660075	10.2113969332	10.7363953725
C33	5.4101714932	4.5187564248	10.6192683876
C34	11.0101525900	8.6049385805	9.3520606290
C35	8.2458745015	11.1293386218	9.4117348498
C36	5.3134821014	7.5175777340	8.3665237419
C37	9.0784296721	5.5177950095	8.5927535808
H38	9.8315701977	8.8832985484	15.7985795809
H39	10.2146213001	7.1495560856	15.6479931467
H40	11.2614262113	8.3664412826	14.8377771653
H41	6.4522131135	11.4658563582	14.5691563575
H42	5.6436261714	10.0484177763	15.3219169182

H43	7.3454242194	10.3985306709	15.7123192050
H44	3.9433032965	8.5457049912	14.1461397015
H45	3.4334132940	6.8916230831	13.6820136934
H46	4.4777001058	7.1408094192	15.1271015715
H47	6.2542883331	4.3309825441	14.0856733172
H48	7.8741726046	3.6050007522	13.8486390478
H49	7.5487683477	4.6359376373	15.2815110227
H50	10.8461842755	5.2674902678	13.4679106755
H51	11.0809352347	4.5907893332	11.8274381919
H52	12.0142055820	6.0412674436	12.3435208390
H53	9.4194878099	12.1172338343	12.6537199363
H54	9.8141109157	11.0113527577	14.0068806094
H55	10.9258436994	11.1396020406	12.6060105946
H56	4.5150513963	11.2077311730	11.1783362159
H57	4.9420822825	10.2638223512	9.7140955869
H58	3.5862588844	9.7259916326	10.7657734684
H59	4.3565631653	4.7804835859	10.5163115150
H60	5.8557779651	4.2845634669	9.6512600605
H61	5.5369672792	3.6752037954	11.2989880499
H62	11.5600711635	7.6620802397	9.3762401054
H63	10.6843942202	8.8333122348	8.3373782485
H64	11.6283987130	9.4093201466	9.7527413739
H65	7.6967986504	12.0244756196	9.7070970436
H66	9.2914733310	11.2031591965	9.7187194581
H67	8.1714059450	10.9641301686	8.3358654368
H68	5.4052517684	6.4544500178	8.1334152422
H69	4.2760928511	7.7642477573	8.5934488087
H70	5.6871557142	8.1215691089	7.5390595255
H71	10.1291481684	5.4442544948	8.8794603649
H72	8.6309562598	4.5238246568	8.5683710849
H73	8.9756931714	6.0206852760	7.6301923140

Table S9. Geometry Optimized Coordinates of **[$V^{IV}_2V^{V}_3Fe^{III}Cl]^{1+}$**

Fe1	7.4570144525	7.5459746700	13.9387165471
V2	9.9225552542	8.2158983144	12.0617568238
V3	7.2470263158	10.0857648601	12.0104663242
V4	5.4784975914	7.4103282457	11.4322792960
V5	8.1272758310	5.5718562317	11.5022184130
V6	7.9704854063	8.1597519351	9.4939976172
Cl7	7.1557838927	7.3697349150	16.2066011691
O8	7.6918163147	7.8625763011	11.8106964834
O9	9.4370876085	8.0135016536	13.8967705896
O10	7.0904965853	9.6138839449	13.8103347054
O11	5.4854406347	7.3948174572	13.3082128722
O12	7.7853064739	5.6727192256	13.4780215640

O13	9.9788790429	6.3295736839	11.8738767181
O14	9.2051899128	10.0688440481	12.2339571426
O15	5.4802678812	9.4259418857	11.6037243177
O16	6.0957597972	5.6538278095	11.2669940287
O17	9.8480235091	8.4493940168	10.1856196261
O18	7.6036631182	10.0521737073	10.1441998288
O19	6.0044615987	7.7817091843	9.6257462201
O20	8.2991494469	6.2232721459	9.6257175168
O21	11.4529028017	8.5174463118	12.3062166486
O22	6.9907131126	11.6392543929	12.1584462699
O23	3.9391579368	7.1865594026	11.1605858900
O24	8.4190198855	4.0203737540	11.3192070567
O25	8.1350854476	8.4607929987	7.9412396772
C26	10.3188609060	7.9242274374	15.0619010689
C27	6.6181979032	10.4542561179	14.9119968969
C28	4.3023168395	7.6237556507	14.1422952991
C29	7.2171358008	4.5400515457	14.2105992643
C30	10.9972781971	5.4603047199	12.4765004329
C31	9.8414344804	11.0210352830	13.1473395907
C32	4.5754803578	10.2162598613	10.7584800576
C33	5.3653077763	4.4840064468	10.7604131063
C34	10.9985618953	8.7808929355	9.3356695655
C35	8.1983383767	11.1854889749	9.4336091346
C36	5.2899698683	7.2742079538	8.4500746568
C37	9.2686321933	5.5808437674	8.7329802703
H38	9.9461178337	8.5996388390	15.8336802879
H39	10.2990032075	6.9012498583	15.4425989270
H40	11.3299652326	8.2022555771	14.7563688551
H41	6.6489923996	11.4984462501	14.5935567124
H42	5.5935050991	10.1666025232	15.1571145761
H43	7.2576474087	10.2850573970	15.7787153300
H44	4.0072537143	8.6728625813	14.0532640285
H45	3.4960681517	6.9796419721	13.7841026450
H46	4.5641825124	7.3868719141	15.1736313043
H47	6.1410570950	4.4815064961	14.0183835405
H48	7.7077024710	3.6271888564	13.8652027833
H49	7.3918371866	4.6912089081	15.2772206335
H50	10.6914161395	5.2138744096	13.4957691818
H51	11.0580297526	4.5510338623	11.8767238226
H52	11.9513099527	5.9915631414	12.4772247811
H53	9.4648179085	12.0195216918	12.9158911538
H54	9.5891897390	10.7520168615	14.1766082731
H55	10.9213190750	10.9710365723	12.9963897085
H56	4.5385008841	11.2329850141	11.1545851259
H57	4.9703524772	10.2166605148	9.7397684665
H58	3.5896212594	9.7496640088	10.7918428646
H59	4.3087972704	4.7434086995	10.6738987398

H60	5.7827926246	4.2065564966	9.7910044909
H61	5.5062598627	3.6650249194	11.4675400409
H62	11.7326586973	7.9753662182	9.4087000827
H63	10.6440957375	8.8844725628	8.3101494017
H64	11.4385136509	9.7160387094	9.6890122946
H65	7.7108302925	12.1021802173	9.7723041069
H66	9.2663995191	11.2202950186	9.6642916497
H67	8.0461115320	11.0296578334	8.3646775184
H68	5.6301431224	6.2574277915	8.2398810088
H69	4.2193950616	7.2813710685	8.6663562130
H70	5.5234388319	7.9263092377	7.6072952653
H71	10.2871623094	5.8247513932	9.0494712508
H72	9.1152319840	4.5009051745	8.7815270915
H73	9.0952502704	5.9476945481	7.7187638613

Table S10. Geometry Optimized Coordinates of **[$V^{IV}_3V^{V}_2Fe^{III}Cl$]**

Fe1	7.4904148848	7.6583803467	13.9508882216
V2	9.9579097060	8.2822866899	12.0284998676
V3	7.2728038762	10.1495919128	11.9460738223
V4	5.4445696283	7.4644230678	11.5136173368
V5	8.1363759166	5.6097792909	11.5812767766
V6	7.9266492556	8.0896157875	9.4853290740
Cl7	7.2979382457	7.4307519063	16.2801812486
O8	7.6969665372	7.8554391955	11.8064507522
O9	9.4332160678	8.0176087951	13.9400114369
O10	7.0900002395	9.6959672620	13.7770295480
O11	5.5947127382	7.3368701320	13.5031972606
O12	7.8750230337	5.6628194954	13.4509897936
O13	9.9323392255	6.2511281484	11.8453359469
O14	9.1664421654	10.1560629402	12.1982227180
O15	5.4930076448	9.5020925160	11.6516077527
O16	6.2227219766	5.5843863719	11.3619757087
O17	9.7965526380	8.4207501020	10.0526809956
O18	7.5747145810	10.0055374069	10.0534731537
O19	5.9809018960	7.6792386002	9.6148578060
O20	8.2808357383	6.1080183991	9.7342016766
O21	11.5153694849	8.5982350594	12.1998666944
O22	6.9761887957	11.7065922614	12.0371287861
O23	3.8786685350	7.1910895795	11.3522225315
O24	8.4265036566	4.0585296279	11.4067414084
O25	8.0533941103	8.2354155472	7.9002478557
C26	10.3277510303	8.0372844844	15.0837012942
C27	6.0912365609	10.3304754378	14.6294080689
C28	4.4626793444	6.9740343560	14.3392200277
C29	7.2476711064	4.5675476976	14.1790438611
C30	10.9002058043	5.4552165373	12.5932195042

C31	9.7903308694	11.0992426846	13.1220847120
C32	4.5394379719	10.3004374594	10.8873758754
C33	5.6049684531	4.6662682191	10.4101242702
C34	10.9622299350	8.3019885117	9.1944889177
C35	8.2754299351	11.0691258401	9.3359747238
C36	5.0662728790	7.7426544327	8.4904886763
C37	9.0767524458	5.3440168877	8.7831458232
H38	9.9716576383	8.7723180520	15.8103944140
H39	10.3351612623	7.0536021088	15.5620213202
H40	11.3319000884	8.2980932372	14.7390297349
H41	6.1818166113	11.4149460467	14.5192152409
H42	5.0975509459	10.0038313707	14.3079255477
H43	6.2791471377	10.0230033454	15.6590304002
H44	3.6509282163	7.6892825807	14.1757170088
H45	4.1121881347	5.9741267694	14.0640828312
H46	4.7768999869	6.9873258229	15.3848994836
H47	6.1989940286	4.4978861270	13.8742628835
H48	7.7687295642	3.6399490298	13.9240799479
H49	7.3215610651	4.7803684700	15.2463097524
H50	10.5767529143	5.3849552089	13.6357608154
H51	10.9432616346	4.4565414986	12.1503905560
H52	11.8697494877	5.9529128384	12.5270258095
H53	9.4352880135	12.1051932311	12.8826926368
H54	9.5004517151	10.8344065867	14.1430709612
H55	10.8721548678	11.0252406288	12.9974982391
H56	4.4514203708	11.2827472449	11.3595243944
H57	4.9124730835	10.4159915192	9.8658436527
H58	3.5808592866	9.7783776579	10.8926603149
H59	4.5229661054	4.7146099953	10.5492449652
H60	5.8754459575	4.9751518996	9.3963074913
H61	5.9792079712	3.6578020517	10.6072712921
H62	11.4166514776	7.3111253326	9.2996127770
H63	10.6503217272	8.4593505437	8.1584530055
H64	11.6948066009	9.0581572643	9.4909600833
H65	7.8229860262	12.0262533116	9.6086721529
H66	9.3293712299	11.0511663254	9.6276188050
H67	8.1726363723	10.8755569503	8.2667070977
H68	4.2733051505	7.0010366220	8.6245682736
H69	4.6134617800	8.7376895022	8.4253466856
H70	5.6233912996	7.5427024760	7.5718462372
H71	10.1346518287	5.4204056578	9.0502023096
H72	8.7663475430	4.2964909411	8.8304321730
H73	8.9065753083	5.7560949230	7.7869063465

Table S11. Geometry Optimized Coordinates of **[$V^{IV}_4V^{V}Fe^{III}Cl]^{1-}$**

Fe1	7.4223055860	7.5513854347	13.8944616513
V2	9.9042505836	8.2312944264	12.0382308539

V3	7.2401359245	10.1460411131	12.0382727531
V4	5.3662757407	7.4566467491	11.4245155234
V5	8.1009136179	5.5813357484	11.4438865872
V6	7.9107996205	8.2204676286	9.4490176858
Cl7	7.3157020987	7.2224000455	16.2804578684
O8	7.7585039794	7.8722781112	11.8092914235
O9	9.4906613784	7.9319518437	13.8667281929
O10	7.1312364440	9.5282281951	13.9389442731
O11	5.5058147238	7.2575252761	13.4151274341
O12	7.8524596140	5.6391179005	13.4300456046
O13	9.9871393460	6.3187312716	11.7860380735
O14	9.2907560149	10.0570367961	12.2601815087
O15	5.4132574625	9.4522728519	11.6606232593
O16	6.1342089181	5.6159405082	11.2208909218
O17	9.8302966057	8.4752370070	10.1454651538
O18	7.5646855123	10.0801380410	10.0775483673
O19	5.9899108043	7.7868198992	9.5383652766
O20	8.2997391778	6.2695886899	9.5683978321
O21	11.4651924830	8.4891314413	12.2640987390
O22	7.0061406248	11.7194042590	12.2615862341
O23	3.8045829375	7.1724622575	11.1991304731
O24	8.4472968554	4.0299330548	11.2131929091
O25	8.1447056003	8.5029549313	7.8839732157
C26	10.3696620398	7.8327415921	15.0086814891
C27	6.6033148754	10.3666404536	14.9900804147
C28	4.4145274323	7.7552164763	14.2323945811
C29	7.1609558505	4.5591532265	14.1078477010
C30	10.9269334370	5.4676723898	12.4891070935
C31	9.8990862074	10.9323589100	13.2425073604
C32	4.5702148755	10.2299091506	10.7599010332
C33	5.3557919640	4.4282534195	10.9547028264
C34	10.9562420312	8.8839709713	9.3321514994
C35	8.2342616016	11.1978995544	9.4442115997
C36	5.4552106309	6.9252082955	8.4961207616
C37	9.3622523918	5.7166217314	8.7514595249
H38	10.0358809047	8.5348267528	15.7774083030
H39	10.3148252757	6.8207608245	15.4192104197
H40	11.3915742817	8.0652948040	14.6897238976
H41	7.2015291322	11.2833258567	15.0559116126
H42	5.5675982700	10.6463664458	14.7670865932
H43	6.6462449803	9.8213188915	15.9366061647
H44	4.2245403489	8.8084216227	14.0007302542
H45	3.5188805970	7.1680059129	14.0020609729
H46	4.6824239649	7.6413655844	15.2867057174
H47	6.0802845155	4.6535523697	13.9530297565
H48	7.5178609235	3.6067260330	13.7004810044
H49	7.3778011364	4.6240364961	15.1777017489
H50	10.5465958645	5.2633963895	13.4945498890
H51	11.0138306213	4.5287193223	11.9354159172
H52	11.8949553020	5.9772340536	12.5423889526
H53	9.5689417420	11.9541336570	13.0364044776

H54	9.5739647618	10.6334480482	14.2442842452
H55	10.9884985187	10.8519339839	13.1626330041
H56	4.6030197192	11.2775041016	11.0756307043
H57	4.9379112789	10.1347729280	9.7346117729
H58	3.5493064933	9.8409285490	10.8304969351
H59	4.3286791266	4.7231320887	10.7217944799
H60	5.7905670123	3.8830019868	10.1094878623
H61	5.3544509186	3.7726403565	11.8334728108
H62	11.7837244377	8.1824983760	9.4816732752
H63	10.6430462514	8.8867897642	8.2857781530
H64	11.2751257569	9.8881974518	9.6316621599
H65	7.6745500621	12.1107402967	9.6732862617
H66	9.2566015093	11.2982432497	9.8273048072
H67	8.2550512504	11.0235040895	8.3638162008
H68	5.9090002214	5.9324895365	8.5667160558
H69	4.3710782655	6.8568672464	8.6306243578
H70	5.6913817911	7.3721409696	7.5250178766
H71	10.3401746090	6.0323681009	9.1325658324
H72	9.2917163414	4.6246921666	8.7848095785
H73	9.2309458552	6.0770394276	7.7255019944

Table S12. Geometry Optimized Coordinates of **[$V^{IV}_5Fe^{III}Cl]^{2-}$**

Fe1	7.5043791735	7.5749814225	13.8742014079
V2	9.9792237297	8.2816269410	12.0125324610
V3	7.2254777050	10.1614158712	12.0301664682
V4	5.3903715123	7.4388161171	11.4744859629
V5	8.1368360245	5.5818118896	11.4538803012
V6	7.8991050274	8.2172164244	9.4293686768
Cl7	7.3091724744	7.2288766168	16.3402165631
O8	7.6961611075	7.8616531703	11.8161853185
O9	9.4803248017	7.9477291374	13.9241387913
O10	7.1001710120	9.5531634388	13.9421131086
O11	5.5385057909	7.2398214723	13.4668155146
O12	7.8729517548	5.6253533138	13.4463832087
O13	10.0035514645	6.2807262390	11.7557986297
O14	9.2331715605	10.1423153089	12.2469054253
O15	5.3803082615	9.4360152202	11.6761477593
O16	6.1377346880	5.5835046112	11.2735538225
O17	9.7952721902	8.4914414098	10.0165080702
O18	7.5423901946	10.0842046036	10.0552099389
O19	5.9534917066	7.7718696993	9.5692043808
O20	8.2704812580	6.2524496992	9.5552305000
O21	11.5581143590	8.5891985843	12.1584408653
O22	6.9055135761	11.7319328783	12.2236120746
O23	3.8175828446	7.1301458980	11.2774240392
O24	8.4382660296	4.0155905383	11.2050151194
O25	8.0155747688	8.4698016103	7.8380507814
C26	10.3632639977	7.7979461352	15.0457789884

C27	6.5466984162	10.3696778949	14.9854162391
C28	4.4567115973	7.7324441728	14.2861768028
C29	7.0933666579	4.5816716243	14.0745156500
C30	10.8797158767	5.4693759107	12.5716553765
C31	9.7891161842	10.9521937910	13.3087003339
C32	4.5253318758	10.1817491349	10.7722215244
C33	5.3933352618	4.4229992016	10.8765888829
C34	10.9255391836	8.7699117954	9.1784357573
C35	8.3208198230	11.1588047723	9.4780682911
C36	5.4047050181	6.9307690016	8.5293014156
C37	9.3420460527	5.7048755831	8.7519761763
H38	10.0565649254	8.4768994488	15.8507183234
H39	10.3164346267	6.7730266270	15.4340539537
H40	11.3868839821	8.0315953203	14.7280156144
H41	7.1503636185	11.2815074745	15.0990402837
H42	5.5202432829	10.6696867186	14.7365016017
H43	6.5510630135	9.8049662118	15.9231756125
H44	4.2494421207	8.7830229748	14.0506924216
H45	3.5586780822	7.1370372156	14.0740820441
H46	4.7373709826	7.6313221163	15.3396706287
H47	6.0360551332	4.6946139837	13.8088229726
H48	7.4675626695	3.6110551412	13.7239672220
H49	7.2054999851	4.6678818591	15.1599597437
H50	10.4283742829	5.2904582059	13.5535413524
H51	11.0312020458	4.5068782224	12.0661353200
H52	11.8378726058	5.9905806594	12.6858410856
H53	9.4476800382	11.9862379915	13.1743269324
H54	9.4542795625	10.5798282120	14.2832526898
H55	10.8831180550	10.9010126187	13.2504448436
H56	4.5375871476	11.2354353673	11.0747711823
H57	4.8922106422	10.0854552572	9.7453204781
H58	3.5100883796	9.7728408083	10.8440184354
H59	4.3329958112	4.6887162224	10.7984596320
H60	5.7530568720	4.0464598591	9.9093119996
H61	5.5168119940	3.6244767903	11.6212497814
H62	11.6863666717	7.9856480298	9.2932760729
H63	10.5965139453	8.8138701321	8.1333282209
H64	11.3772257460	9.7304897895	9.4630037229
H65	7.8013283449	12.1046327931	9.6762335739
H66	9.3157295742	11.1889044758	9.9355647162
H67	8.4043857854	10.9891483841	8.3981289480
H68	5.9240609204	5.9665719832	8.5081826278
H69	4.3376083028	6.7796045753	8.7297695052
H70	5.5434553927	7.4356380274	7.5651301315
H71	10.3126821848	5.9433584563	9.2000349956
H72	9.2197961520	4.6155532423	8.7117526243
H73	9.2766784713	6.1345066579	7.7449378697

Table S13. Geometry Optimized Coordinates of **[$V^{IV}_2V^{V}_3Fe^{III}$]$^{2+}$**

Fe1	22.7410613531	15.5644985993	10.9862176886
V2	21.3451323801	17.7620738074	12.5456939152
V3	21.1580772480	17.5523506599	9.2633830190
V4	24.4480566238	17.2494352232	9.1074486236
V5	24.6404467727	17.5052565973	12.4149527666
V6	23.0934022416	19.7922671648	10.6385026473
O7	22.7833055345	17.3017952538	8.0931645673
O8	21.4620526123	15.7014108165	12.3810101832
O9	21.3277235397	15.6407721239	9.5422740118
O10	21.7628896437	19.6343880444	12.0791753802
O11	20.1700864564	17.6668007582	10.8575879868
O12	21.6043616228	19.3740498474	9.3051577336
O13	20.3166803179	17.9005321183	13.7375545798
O14	20.0386676480	17.5511048643	8.1602312342
O15	22.9084010177	17.4819398825	10.7917976567
O16	23.1397809113	17.5972002407	13.5354845136
O17	24.0989873807	15.3978383218	9.4931169332
O18	24.3228729259	15.6040238778	12.2313315223
O19	24.3924563489	19.1198662362	9.1873531512
O20	25.6183007006	17.3102327791	10.6920377695
O21	24.5033536616	19.3458070026	12.0302791142
O22	25.5061741539	17.0844967148	7.9591949698
O23	25.8285983139	17.4755438211	13.4443568624
O24	23.2840121500	21.3514755146	10.4461395333
C25	22.7603220809	17.8551454183	6.7233512441
C26	20.5672556600	14.7430635045	13.0308343644
C27	21.0980346320	14.6493466574	8.4835625907
C28	20.8125764522	20.7100348691	12.4219048091
C29	18.7666279095	17.2091282704	10.9694629745
C30	20.7785238825	20.4634846374	8.7375428399
C31	23.1299866427	17.0411117358	14.9091323825
C32	24.7080739891	14.2500210600	8.8032912898
C33	25.4027797951	14.6135586208	12.3274761382
C34	25.2037697420	20.0879793015	8.4174049775
C35	26.9706802428	17.9000343339	10.5836467402
C36	24.8259675330	20.3697716341	13.0547203005
H37	22.7956848077	18.9453045254	6.7869390521
H38	23.6272889382	17.4723406792	6.1830333334
H39	21.8344448110	17.5349908232	6.2434731028
H40	21.1633041379	13.9279640319	13.4493304441
H41	20.0258301039	15.2549852006	13.8276230226
H42	19.8634804533	14.3473607127	12.2936730189
H43	20.1425535905	14.8727105315	8.0042818309
H44	21.9072343284	14.7094088512	7.7527698576
H45	21.0607347539	13.6572873068	8.9395402646
H46	19.8829584859	20.5723284532	11.8641136496
H47	20.6087772965	20.6571648464	13.4927774907
H48	21.2698517456	21.6676881467	12.1685094572
H49	18.3745854118	17.5656446895	11.9217136373
H50	18.2002761542	17.6229189514	10.1337938363
H51	18.7564485662	16.1180537659	10.9298250045

H52	21.4405529972	21.2897049379	8.4793018574
H53	20.2568872907	20.0818513789	7.8588225711
H54	20.0605584957	20.7798858809	9.4964320504
H55	22.3062287384	17.5087047491	15.4480289844
H56	22.9835069867	15.9615371370	14.8334584107
H57	24.0873832852	17.2672883928	15.3805082873
H58	24.3449842209	13.3266937854	9.2593108975
H59	24.4310108002	14.2803030872	7.7477243240
H60	25.7935976683	14.3122742618	8.9010072512
H61	26.0601551082	14.7164051663	11.4614720927
H62	25.9599291954	14.8033097289	13.2470180256
H63	24.9577765394	13.6166131909	12.3637480403
H64	25.7834265037	20.6877893443	9.1199838248
H65	25.8549881897	19.5329691911	7.7413039008
H66	24.5243668986	20.7360681327	7.8619189040
H67	27.4240690964	17.5456703477	9.6570427457
H68	26.8726844290	18.9873436990	10.5796732408
H69	27.5524705647	17.5752559549	11.4471248593
H70	24.0048334488	20.3971346925	13.7747611836
H71	25.7580238967	20.0819264861	13.5432839398
H72	24.9255093259	21.3275779130	12.5444057819

Table S14. Geometry Optimized Coordinates of **[$V^{IV}_3V^{V}_2Fe^{III}$]$^{1+}$**

Fe1	22.7409798999	15.5703537214	11.0107338617
V2	21.3357386591	17.8561031874	12.4730055343
V3	21.2141444709	17.4630954742	9.1800343450
V4	24.5254580456	17.2408518153	9.1062053982
V5	24.6348035600	17.4935037120	12.4094502498
V6	23.1191747751	19.8006280946	10.5675257541
O7	22.8986948972	17.2595684589	8.0594471439
O8	21.3960222456	15.8873409888	12.4392505423
O9	21.3576200942	15.4541447695	9.6515220233
O10	21.7199143344	19.6441636099	12.0579334211
O11	20.1922320105	17.7063001499	10.9337735583
O12	21.6454654415	19.3891752459	9.2837210655
O13	20.2519565060	17.9948689332	13.6094480018
O14	20.0515110057	17.4035416471	8.0977164353
O15	22.9123467673	17.4862701886	10.8068748880
O16	22.9343014621	17.6933966119	13.5230596691
O17	24.1528922554	15.4126531690	9.5116171244
O18	24.1982995871	15.4730205142	12.2840651846
O19	24.4371438853	19.1362907593	9.1749001673
O20	25.6579244483	17.3007588485	10.6677547008
O21	24.4729725468	19.4171605163	11.9850392113
O22	25.6007988147	17.1006396793	7.9640745969
O23	25.7875453927	17.4441360610	13.5025929601
O24	23.2613068730	21.3734745099	10.3937604548
C25	22.8789384962	17.8650286330	6.7192269620
C26	20.5274641061	14.9247713582	13.0907517434

C27	21.1193390922	14.4647050956	8.6069874636
C28	21.0276643676	20.8065436009	12.6349628465
C29	18.8201967304	17.1888147714	10.9977748266
C30	20.5750166749	20.3647459242	9.0570124165
C31	22.9026218908	17.0526204762	14.8440683356
C32	24.6675502361	14.2671885505	8.7671245016
C33	25.2999672714	14.5211958578	12.3821669463
C34	25.2100170169	20.1032330865	8.3850311631
C35	26.9944891562	17.9117559009	10.6116225089
C36	24.6691822008	20.3907625776	13.0649364290
H37	22.8482173284	18.9524323904	6.8217215265
H38	23.7793250154	17.5534964185	6.1853274915
H39	21.9825101572	17.5135263034	6.2060040450
H40	21.1458489242	14.1688179843	13.5846450104
H41	19.9156790379	15.4402582793	13.8345514231
H42	19.8823636065	14.4465926428	12.3472627793
H43	20.1166145163	14.6245085747	8.2037764033
H44	21.8550024248	14.5810840343	7.8067025771
H45	21.1867153147	13.4621668765	9.0403542888
H46	19.9893515610	20.7984924234	12.2960195713
H47	21.0668769139	20.7304021239	13.7232741764
H48	21.5341406840	21.7080257872	12.2908135271
H49	18.3968565949	17.4359761408	11.9733834151
H50	18.2438554171	17.6579916772	10.1986032437
H51	18.8436593455	16.1073869261	10.8488192857
H52	21.0204229788	21.3616863346	9.0317385221
H53	20.0972719909	20.1363523313	8.1016764076
H54	19.8337488555	20.3027036944	9.8591863150
H55	22.0427947805	17.4383935841	15.3958467140
H56	22.8182904274	15.9726561311	14.7043280107
H57	23.8334735073	17.2932645182	15.3596039254
H58	24.3138043105	13.3457169797	9.2363722324
H59	24.3142999164	14.3155799116	7.7340518452
H60	25.7599360240	14.2929326644	8.7831903471
H61	25.9623265239	14.6320062489	11.5191789650
H62	25.8525856881	14.7318980211	13.3005401247
H63	24.8941706472	13.5057837211	12.4228650094
H64	25.7937743707	20.7219441400	9.0687423892
H65	25.8623310163	19.5533380726	7.7047800423
H66	24.5124327904	20.7328317858	7.8300469669
H67	27.4720474560	17.6169927268	9.6748017286
H68	26.8880751207	18.9976537889	10.6679688978
H69	27.5644922834	17.5487337961	11.4682643331
H70	23.8362642878	20.3351095861	13.7719538501
H71	25.6036775012	20.1530535598	13.5778177219
H72	24.7212877788	21.3874074316	12.6206361755

Table S15. Geometry Optimized Coordinates of **[$V^{IV}_4V^{V}Fe^{III}$]**

Fe1	22.7373250311	15.5995511920	11.0088823901

V2	21.3040429941	17.8309568077	12.5016201805
V3	21.1727249092	17.4760304913	9.1535326371
V4	24.5270566038	17.1509571755	9.0972730540
V5	24.6593109122	17.5012823528	12.4429670181
V6	23.1238499209	19.7328162218	10.5608901940
O7	22.8251842944	17.2354516356	8.0343147798
O8	21.3957176919	15.8138467061	12.4233463400
O9	21.2970511406	15.5125506100	9.6237769030
O10	21.8055767661	19.7207807656	11.9592559274
O11	20.1552930572	17.7437044302	10.8584271993
O12	21.6874127756	19.4418980745	9.3019955237
O13	20.2266876714	18.1213078049	13.6423084680
O14	20.0160894270	17.5174439398	8.0519031433
O15	22.9215627064	17.5099291503	10.7933503168
O16	23.0070055850	17.7313888064	13.5517382731
O17	24.0728383370	15.2446904992	9.5871267663
O18	24.2204875256	15.5317188973	12.3369412740
O19	24.3783171256	19.1806136157	9.2374922918
O20	25.6852017829	17.2665538069	10.7340621652
O21	24.4567096292	19.4673347495	11.9309001202
O22	25.6186574966	16.9419780081	7.9511312593
O23	25.8205554498	17.5623892609	13.5381015728
O24	23.2338197216	21.3066994067	10.4184928165
C25	22.8534837936	17.9077919534	6.7390573913
C26	20.5034021572	14.8701702220	13.0548245375
C27	21.2205793610	14.5599952712	8.5272361206
C28	21.1615564382	20.8690700095	12.5788930971
C29	18.8334752676	17.1327031807	10.9446731237
C30	20.6180092189	20.4175739739	9.1184968918
C31	22.9728212750	17.0444045497	14.8401829726
C32	24.6285736668	14.1120474017	8.8799709094
C33	25.3379322035	14.6007956365	12.3132755856
C34	25.1632633364	20.1123260450	8.4361163951
C35	26.9758928395	17.9383381735	10.6276385835
C36	24.6599206553	20.4430533194	12.9976718542
H37	22.8656286060	18.9939713195	6.8771096462
H38	23.7516621608	17.5884720039	6.2028957316
H39	21.9567707628	17.6250182269	6.1809432420
H40	21.0886877625	14.1386470780	13.6236474304
H41	19.8395436097	15.4084878204	13.7354830200
H42	19.9060775200	14.3511746803	12.2963258340
H43	20.2233480677	14.6293669857	8.0825625231
H44	21.9760818182	14.7959616131	7.7733196019
H45	21.3766372270	13.5469291429	8.9147545785
H46	20.0858474505	20.8164755944	12.3935099863
H47	21.3424287060	20.8355296927	13.6560170715
H48	21.5797843829	21.7816845110	12.1470431038
H49	18.3907811854	17.3943274105	11.9097517739
H50	18.2184853820	17.5232053282	10.1286949801
H51	18.9159665999	16.0462953791	10.8466313999
H52	21.0532711440	21.4207683660	9.1328430093

H53	20.1382924076	20.2162345092	8.1582370517
H54	19.8884770410	20.3039761695	9.9248780529
H55	22.0723244033	17.3591697296	15.3748750042
H56	22.9608263283	15.9620391438	14.6827702070
H57	23.8669518966	17.3250527754	15.4039836214
H58	24.2409502825	13.1810688698	9.3085444993
H59	24.3592335552	14.1628291794	7.8202489062
H60	25.7196783998	14.1248486831	8.9674930333
H61	25.9883166059	14.8239948653	11.4638017113
H62	25.8956953340	14.7133755213	13.2475221532
H63	24.9545049347	13.5768931387	12.2392685543
H64	25.7743496404	20.7300578569	9.0989155665
H65	25.7982527362	19.5343314393	7.7634665999
H66	24.4858782376	20.7514694494	7.8640671000
H67	27.4574112337	17.6177654071	9.6994695220
H68	26.8378597231	19.0244443373	10.6233126160
H69	27.5869662948	17.6563041833	11.4894223020
H70	23.8732056794	20.3180892925	13.7466311221
H71	25.6344849665	20.2496053547	13.4506191513
H72	24.6238840029	21.4469006659	12.5649905749

Table S16. Geometry Optimized Coordinates of **[$V^{IV}_5Fe^{III}]^{1-}$**

Fe1	22.7218772637	15.6098987086	11.0227731708
V2	21.3024819123	17.8748051773	12.5028982443
V3	21.1659789484	17.4946618114	9.1455935127
V4	24.5295078585	17.1420089886	9.0875230168
V5	24.6627984830	17.5192155026	12.4440820591
V6	23.1503995320	19.7997855236	10.5313138277
O7	22.8161023535	17.2121287790	8.0213261130
O8	21.3807973752	15.8237744636	12.4423661509
O9	21.2715038055	15.5007179603	9.6403768676
O10	21.7734823795	19.7390249897	11.9946205583
O11	20.1381878959	17.7483822434	10.8591483342
O12	21.6441399715	19.4231561615	9.2409207533
O13	20.2173515982	18.1092786230	13.6608928768
O14	20.0021583156	17.4677088551	8.0385317826
O15	22.9138515046	17.4927486548	10.8010233397
O16	23.0075187485	17.7455816656	13.5671871029
O17	24.0495318708	15.2088684663	9.6130395591
O18	24.1990016640	15.5174899525	12.3653556317
O19	24.4530802972	19.1195814360	9.1477407565
O20	25.6921064186	17.2386893953	10.7348968165
O21	24.5262480393	19.4530379820	11.9640478356
O22	25.6136679039	16.8226213804	7.9470058966
O23	25.8322008817	17.5117388980	13.5442207758
O24	23.3276534755	21.3789473887	10.3111550552

C25	22.8549911642	17.9490651130	6.7663862172
C26	20.4970435463	14.8975898458	13.0887660362
C27	21.2288130059	14.5669156238	8.5344954396
C28	21.1429448392	20.8856085449	12.6017417833
C29	18.8569084068	17.0717124771	10.9477493167
C30	20.5457654544	20.3736439234	9.1875016461
C31	22.9637327798	17.0197442913	14.8252886549
C32	24.6048644273	14.0798492069	8.9202415854
C33	25.3199641938	14.6022687222	12.3148232807
C34	25.2573792997	20.0349527073	8.3756880468
C35	26.9469627184	17.9660364336	10.6208952693
C36	24.6216056800	20.4053021156	13.0586880486
H37	22.8362648132	19.0247631634	6.9658145693
H38	23.7720695007	17.6782859294	6.2324797593
H39	21.9749648206	17.6726497173	6.1755510752
H40	21.0791865095	14.1735202196	13.6747921664
H41	19.8303310599	15.4470015442	13.7606533285
H42	19.8946575288	14.3574674480	12.3455404953
H43	20.2506667524	14.6507812357	8.0475399678
H44	22.0154472004	14.8047391495	7.8125973180
H45	21.3603634367	13.5438971045	8.9118388333
H46	20.0617486467	20.8606258095	12.4188991273
H47	21.3157782088	20.8736847366	13.6845651608
H48	21.5689352795	21.7953436688	12.1672201731
H49	18.4200186706	17.2713309923	11.9317741249
H50	18.2024717057	17.4605882386	10.1598227234
H51	18.9903661148	15.9945789985	10.8024931380
H52	20.9633008843	21.3846993257	9.2329261755
H53	20.0059746711	20.2259044543	8.2456010611
H54	19.8635442683	20.2089751873	10.0275555781
H55	22.0586213842	17.3164355949	15.3656978491
H56	22.9526975556	15.9411415774	14.6369656032
H57	23.8551191527	17.2792772494	15.4064710838
H58	24.2018988499	13.1459182055	9.3346661380
H59	24.3603765330	14.1327558809	7.8526835872
H60	25.6964131153	14.0779215279	9.0260961410
H61	25.9687445415	14.8523396012	11.4704235107
H62	25.8830483707	14.6947921150	13.2502809020
H63	24.9504829937	13.5730032628	12.2129988200
H64	25.8643008959	20.6567966045	9.0436028601
H65	25.9091850206	19.4627112864	7.7076077590
H66	24.6067179775	20.6912203481	7.7858998166
H67	27.4188093169	17.6989240791	9.6693717442
H68	26.7606529452	19.0433572607	10.6619293265

H69	27.5918451852	17.6773281206	11.4580085147
H70	23.7985072182	20.2509176867	13.7635156407
H71	25.5761822550	20.2472006918	13.5721724531
H72	24.5762848451	21.4171074396	12.6421540006

Table S17. Geometry Optimized Coordinates of **[$V^{III}V^{IV}{}_4Fe^{III}$]$^{2-}$**

Fe1	22.6467536500	15.6544319975	11.0593583029
V2	21.2827711907	17.9423057977	12.5004585825
V3	21.1511739841	17.5172834861	9.0964027267
V4	24.5086086922	17.1281222687	9.1105728520
V5	24.6856014149	17.5029536889	12.4630038749
V6	23.1501967101	19.8038632796	10.5482749912
O7	22.8335499247	17.2229228517	7.9828743123
O8	21.3455189864	15.6437099403	12.4244441768
O9	21.2594678254	15.5374302392	9.5673212369
O10	21.7833461351	19.8679479052	11.9814786611
O11	20.1296420786	17.7235669426	10.7665348987
O12	21.6457562290	19.4505671971	9.2401276725
O13	20.1050013925	17.9879717516	13.7391434035
O14	20.0296058031	17.5302016039	7.9346174736
O15	22.9648241822	17.5152470193	10.7368044506
O16	23.0697466482	17.7147918507	13.5670442736
O17	23.9958489208	15.1972942020	9.6384328972
O18	24.2013955920	15.5240593689	12.3750978601
O19	24.4858040490	19.1090600713	9.1452673711
O20	25.7177286430	17.2031412277	10.7292498362
O21	24.5311044215	19.4405059510	11.9835228171
O22	25.5955384152	16.7742639905	7.9651923765
O23	25.9012608266	17.5066535323	13.5259153750
O24	23.4056823188	21.3785040527	10.2811788971
C25	22.9079894533	17.9678742748	6.7438360803
C26	20.5325311862	14.8284503433	13.2630888030
C27	21.3020064053	14.6065562067	8.4683879198
C28	21.1886993697	21.0007955210	12.6240127443
C29	18.9012020083	16.9705781849	10.9188000915
C30	20.5418312899	20.3898980803	9.2363270402
C31	22.9741555537	16.9591959288	14.7997635015
C32	24.5953698536	14.0551792777	9.0261093570
C33	25.2937122777	14.5892567911	12.2835915489
C34	25.2746900315	19.9956609737	8.3397324687
C35	26.9610988934	17.9328581288	10.6007374652
C36	24.5968753106	20.3746335635	13.0897292901
H37	22.8902761750	19.0443501966	6.9476048704

H38	23.8388663378	17.7000727137	6.2281523894
H39	22.0411955380	17.7054268610	6.1243724683
H40	21.1561232357	14.1789202324	13.8998720251
H41	19.9342442898	15.4984648745	13.8930296088
H42	19.8644191385	14.1912313557	12.6598870435
H43	20.3285465340	14.6224077167	7.9592135514
H44	22.0856584847	14.8889555790	7.7575744872
H45	21.4929973838	13.5916829401	8.8479767346
H46	20.0978191749	20.9791914695	12.4918398011
H47	21.3952024512	20.9750316623	13.7032602825
H48	21.5938325755	21.9275549835	12.1937490410
H49	18.4960318058	17.1930373277	11.9111736200
H50	18.1955799569	17.2738198619	10.1318090263
H51	19.1026746852	15.8968606474	10.8268487853
H52	20.9500381806	21.4067318055	9.2786678888
H53	19.9693204279	20.2480196517	8.3110563109
H54	19.8930430574	20.2082460958	10.0990141873
H55	22.0055060243	17.1885033379	15.2553559261
H56	23.0466058367	15.8854980483	14.5906041289
H57	23.7987154981	17.2539405820	15.4648948669
H58	24.1904900271	13.1288431505	9.4616022460
H59	24.3976675601	14.0518285106	7.9448621200
H60	25.6843817986	14.0672726067	9.1737782032
H61	25.9709945805	14.8699905792	11.4701368706
H62	25.8452567007	14.6019597336	13.2337470614
H63	24.9027115128	13.5758245669	12.1084243617
H64	25.9018387359	20.6310267507	8.9800201035
H65	25.9116570511	19.4067641086	7.6683157810
H66	24.6203344773	20.6500419205	7.7476231395
H67	27.4265882629	17.6691021993	9.6426538664
H68	26.7730136052	19.0115554730	10.6401193627
H69	27.6194346581	17.6532571258	11.4328764707
H70	23.7712804339	20.1907851672	13.7845917666
H71	25.5520361724	20.2291493151	13.6099185155
H72	24.5334400547	21.3935969640	12.6896053831

References:

1. A. D. Bochevarov, E. Harder, T. F. Hughes, J. R. Greenwood, D. A. Braden, D. M. Philipp, D. Rinaldo, M. D. Halls, J. Zhang, R. A. Friesner. Jaguar: A high-performance quantum chemistry software program with strengths in life and materials sciences. *Int. J. Quantum Chem.* **2013**, *113*, 2110–2142.

2. A. V Marenich, R. M. Olson, C. P. Kelly, C. J. Cramer, D. G. Truhlar, Self-consistent reaction field model for aqueous and nonaqueous solutions based on accurate polarized partial charges. *J. Chem. Theory Comput.* **2007**, *3*, 2011–2033.

3. F. Li, S. H. Carpenter, R. F. Higgins, M. G. Hitt, W. W. Brennessel, M. G. Ferrier, S. K. Cary, J. S. Lezama-Pacheco, J. T. Wright, B. W. Stein, M. P. Shores, M. L. Neidig, S. A. Kozimor, E. M. Matson, Polyoxovanadate-alkoxide clusters as a redox reservoir for iron. *Inorg. Chem.*, **2017**, 56, 7065-7080.
4. D. D. Perrin, Ionization constants of inorganic acids and bases in aqueous solution. Pergamon: Oxford, U.K., **1982**.
5. (a) L. E. VanGelder, W. W. Brennessel, E. M. Matson. Tuning the redox profile of polyoxovanadate-alkoxide clusters via heterometal installation: Toward designer redox reagents. *Dalton Trans.* **2018**, *47*, 3698-3704; (b) R. L. Meyer, W. W. Brennessel, E. M. Matson. Synthesis of a gallium-functionalized polyoxovanadate-alkoxide cluster: Toward a general route for heterometal installation. *Polyhedron*, **2018**, *156*, 303-311.
